# *Aster tataricus* L. f.: a review on the botany, phytochemistry, ethnopharmacology, pharmacology, toxicology and comprehensive utilization

**DOI:** 10.3389/fphar.2025.1581505

**Published:** 2025-04-09

**Authors:** Xinyi Zhang, Meiqi Liu, Xiaozhuang Zhang, Lengleng Ma, Shiyi Song, Meitong Pan, Shumin Huang, Weichao Ren, Wei Ma

**Affiliations:** College of Pharmacy, Heilongjiang University of Chinese Medicine, Harbin, China

**Keywords:** *Aster tataricus* L.f., phytochemistry, ethnopharmacology, pharmacological activity, toxicology, comprehensive utilization

## Abstract

*Aster tataricus* L.f. (*A.tataricus*), a perennial herb of the genus Aster in the Asteraceae (Compositae) family. It is associated with a spicy, bitter, and warm nature and belongs to the Lung Meridian. The medicinal parts of *A.tataricus* are flowers, leaves, roots, and rhizomes. *A.tataricus* was first recorded in *Shuo Wen* under the alias “Zi Wan” (茈菀). Traditionally, it is utilised to clear lung qi, promote fluid flow, calm adverse-rising energy, relieve cough, resolve sputum, and regulate secretions. However, it is worth noting that *A.tataricus* has certain hepatotoxicity. Modern pharmacology indicates that *A.tataricus* can be used to treat various diseases, including those of the respiratory and urinary systems. In this review, all available information on *A.tataricus* was collected via academic databases such as PubMed, SciFinder Scholar, CNKI, iPlant, Google Scholar, Web of Science, GBIF, and Masterpieces of Traditional Chinese Medicine. To date, more than 200 metabolites have been isolated and characterized from *A.tataricus*, including terpenoids, flavonoids, polypeptides, and others. These compounds demonstrate a wide range of pharmacological activities, such as anti-inflammatory effects, antitussive and bronchodilatory properties, anticancer activity, antioxidant effects, treatment of osteolytic disorders, management of urinary system diseases, alleviation of acute lung injury, and enhancement of memory. Meanwhile, the different polarity extracts of *A.tataricus* also exhibit some toxicological characteristics, the astin has a similar structure to that of cyclochloridine, the hepatotoxic metabolite of penicillin; its saponins also have hemolytic effects. However, there are currently few studies on the toxicology of *A.tataricus*. Further in-depth research is needed to explore the potential mechanisms underlying the toxicity of *A.tataricus*. The toxicity of *A.tataricus* can be reduced through compatibility and processing, but this aspect has received little discussion and further research on quality standardization is needed. To ensure the sustainable development of *A.tataricus*, we have also summarized its artificial cultivation techniques. Shionone and astin are the characteristic components of *A.tataricus*. Their pharmacological effects have been deeply studied, but the research on other metabolites is relatively scarce. Therefore, this article focuses on botany, artificial cultivation, phytochemistry, ethnopharmacology, pharmacology, toxicology, and comprehensive utilization of *A.tataricus*. Discuss the future research prospects and existing problems of *A.tataricus*, and provide references for further research on *A.tataricus* and the establishment of quality control standards.

## 1 Introduction

**FIGURE 1:**
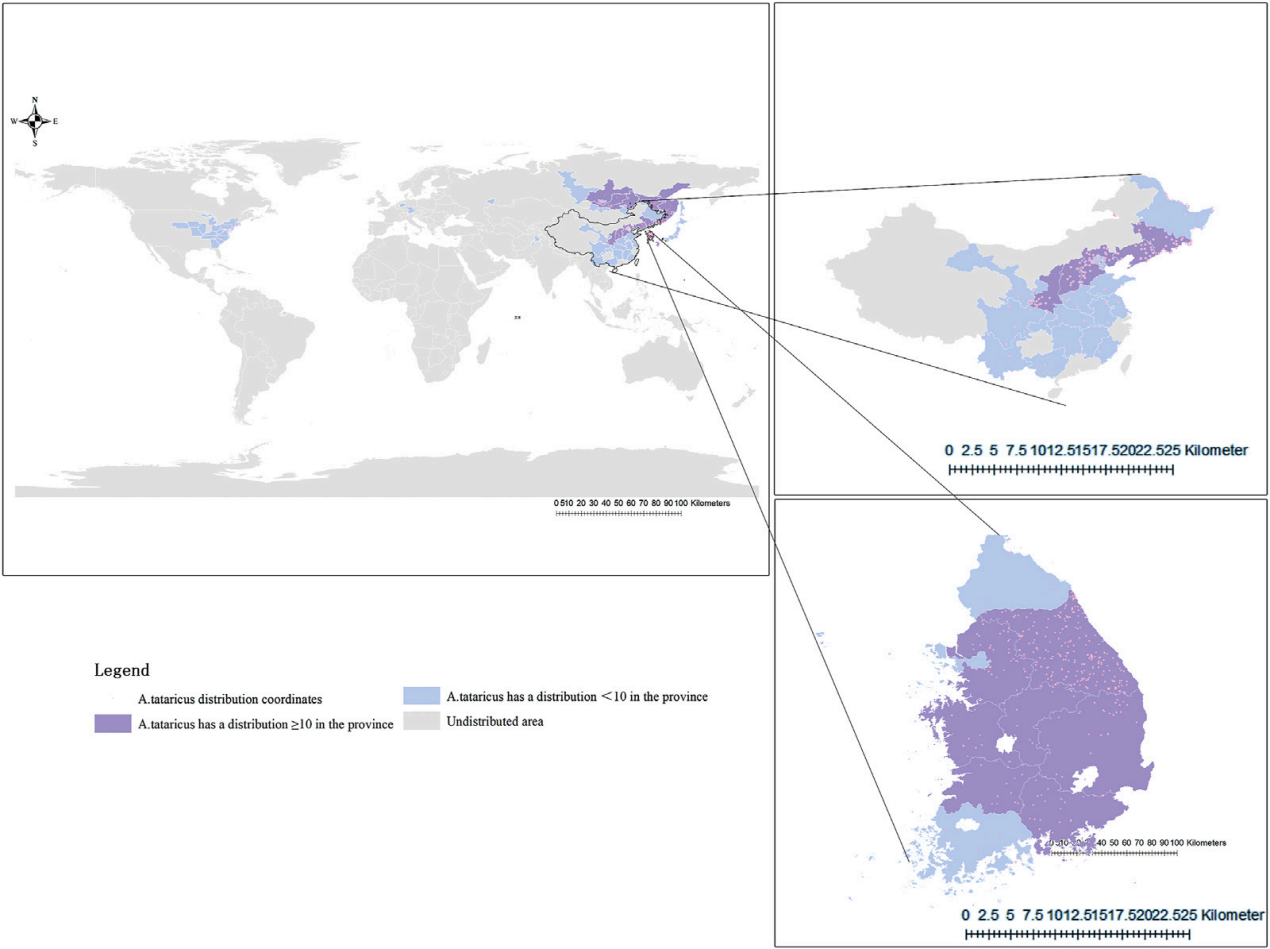
Distribution map of *A.tataricus*. Orange dots represent the distribution position of *A.tataricus.* Purple-shaded areas represent regions where *A.tataricus* is distributed across more than 10 coordinates. Blue-shaded areas represent regions where *A.tataricus* is distributed across less than 10 coordinates, and grey-shaded areas represent regions where *A.tataricus* is not distributed.

According to The *Compendium of Materia Medica*, *Aster tataricus* L.f. (*A.tataricus*) is termed “*Ziwan*” (紫菀) due to its soft, purple roots (K.D, 2023). It is a perennial herb of the genus Aster in the Asteraceae (Compositae) (Yang, 2003). It is also known as China regional common names such as *Huan Hun Cao(*还魂草), *Qing Wan(*青菀), and *Shan Ma Lan (*山马兰) (Li et al., 2010). In temperate regions of America, Europe, and northern Asia, *A.tataricus* is an important cash crop with high ornamental value. Its ability to attract bees and butterflies makes it a popular flower in Europe and America (K.D, 2023). In some Asian countries, young seedlings, tender stems, and leaves harvested from May to June are consumed as vegetables (Ahn et al., 2018). Additionally, *A.tataricus* is used in the production of essential oils (Choi, 2012), pesticides (Rhee et al., 1981), antioxidants in edible oils (Chen et al., 2012), and other products. The plant is distributed across China, Korea, Japan, the northern United States, and eastern Siberia in Russia (Figure 1). It thrives in shady, humid areas at altitudes of 400–2,000 m (Wan et al., 2016). The earliest application of *A.tataricus* can be traced back to the *Shennong Materia Medica*. It is regarded as an important medicine for lung disease, as it moistens the lungs and relieves cough and phlegm. In recent decades have shown that in addition to eliminating phlegm and relieving cough, Extensive biological testing has demonstrated that *A.tataricus* exhibits a wide range of pharmacological properties, such as anti-inflammation (Wu et al., 2023), treating acute lung injury (Song et al., 2022), anti-tumor (Schafhauser et al., 2019), and alleviating osteoporosis (Lee et al., 2023b; Su et al., 2022). Especially, its effect in relieving cough and phlegm has lasted for more than 2000 years (Dong et al., 2024). Traditional medicine practitioners believed that the expectorant and antitussive role of *A.tataricus* is ascribed to the tropism of taste. Different from traditional medicine, modern medical practitioners believe that *A.tataricus* plays a role by reducing inflammatory factors (Deng et al., 2023) and relaxing bronchial smooth muscle (Peng et al., 2016a). Over the past few decades, various nutrients and active compounds have been isolated from *A.tataricus*, including amino acids, essential trace elements, terpenoids, flavonoids, polypeptides, organic acids, and volatile oils (Han et al., 2023). Among these, terpenoids are the most abundant and extensively studied components (Huang et al., 2008). Shionone, a characteristic terpenoid, is present throughout the plant and serves as a quality marker of *A.tataricus* according to the *Pharmacopoeia of the People’s Republic of China* (*ChP*) (Wu et al., 2003; Yang et al., 2024). Studies have shown that polypeptides have obvious anticancer function, but it has a similar structure to that of cyclochloridine, the hepatotoxic metabolite of penicillin (Peng et al., 2016b).

Owing to the remarkable therapeutic efficacy of *A.tataricus*, its market demand has steadily increased over recent years. Consequently, expanding production capacity has become a critical priority. This review provides an in-depth examination of the botany, phytochemistry, ethnopharmacology, pharmacological activities, and toxicology of *A.tataricus*. Additionally, it offers the first comprehensive summary of cultivation techniques and integrated utilization strategies for this species. Furthermore, this study elucidates the “double-edged sword” nature of cyclic peptides—highlighting both their therapeutic benefits and potential toxicity—which serves as a foundational reference for ensuring safe usage and guiding structural modifications in future research, thereby enhancing the developmental potential of *A.tataricus*.

## 2 Materials and methods

### 2.1 Identification and selection of studies

The initial phase of our analysis involved systematically assessing all studies identified through keyword searches pertaining to *A.tataricus*. Following the removal of duplicate entries, we conducted a preliminary review of titles and abstracts to evaluate their relevance based on the established inclusion criteria. For studies that satisfied these criteria, a detailed examination was performed, encompassing a thorough analysis of the full text and an in-depth review of the reference lists to ensure a comprehensive understanding of the relevant literature.

### 2.2 Search strategy

We identified the studies independently using the following keywords: *Aster tataricus*, *A.tataricus* extract, *Fan hun cao*, *Zi wan*. In addition, reported pharmacological activities and phytochemical compositions were searched as keywords. This study only includes results found before March 2025 (without time restrictions before this date). The search was carried out in the electronic bibliographic databases, including PubMed (https://pubmed.ncbi), CNKI (http://www.cnki.net), Baidu Scholar (https://xueshu.baidu.com/), Google Scholar (http://scholar-xm.top) and Traditional Chinese Medicine Baodian Network (www.ZYBD.com).

### 2.3 Inclusion and non-inclusion criteria

Our inclusion criteria encompassed all experimental studies investigating various aspects of *A.tataricus*, including its botany, phytochemistry, ethnopharmacology, pharmacology, toxicology, and comprehensive utilization, irrespective of the extraction methods employed. Additionally, we incorporated Chinese dissertations and theses that detailed the properties of *A.tataricus*. Studies performed in humans, editorials, conference abstracts, the contents of dissertations that have been published in scientific journals, and conference proceedings were also excluded.

### 2.4 Search results

**FIGURE 2:**
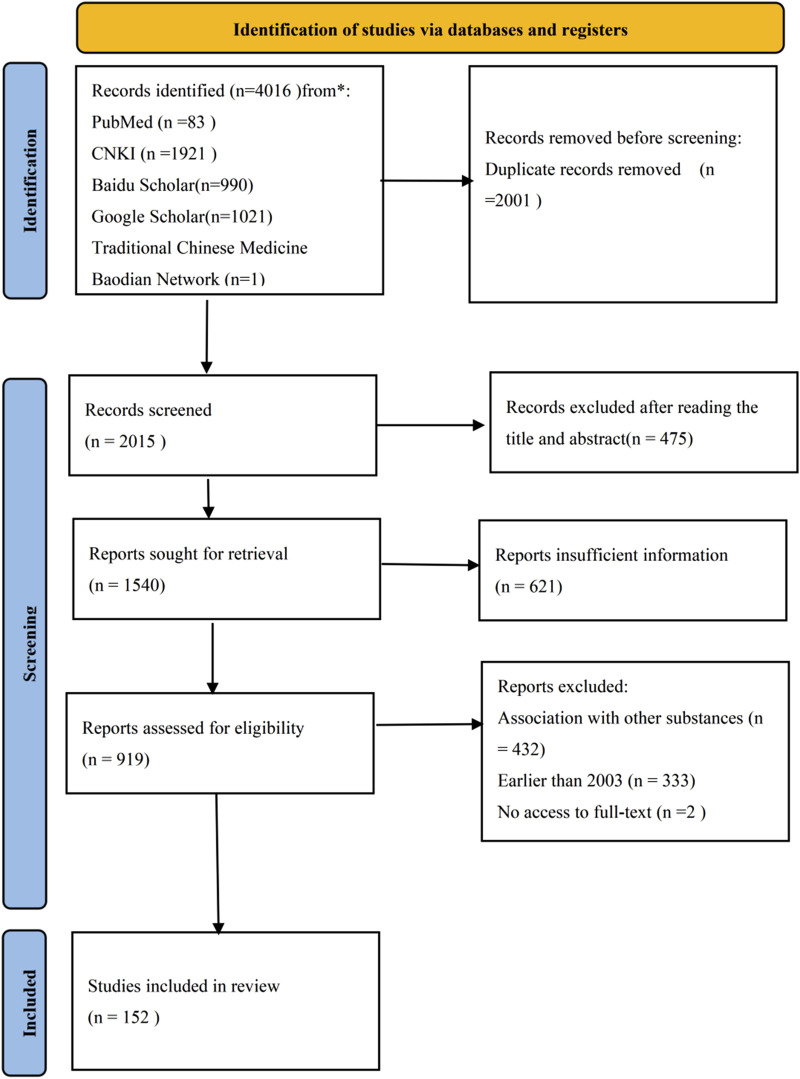
Flowchart of included studies in the review.

The preliminary search yielded a total of 4016 records, distributed across various databases as follows: 83 from PubMed, 1921 from CNKI, 990 from Baidu Scholar, 1021 from Google Scholar, and one from the Traditional Chinese Medicine Baodian Network. After removing 2001 duplicate entries, we conducted a thorough review of titles and abstracts, which led to the exclusion of an additional 475 reports. This left 2015 studies for further evaluation. Upon detailed examination of the full texts, 767 studies were excluded: 432 for involving *A.tataricus* in combination with other substances, 333 for being published prior to 2003, and 2 due to unavailability of the full text. Through this rigorous screening process, a final set of 152 articles was selected for inclusion (Figure 2).

### 2.5 Others

**FIGURE 3:**
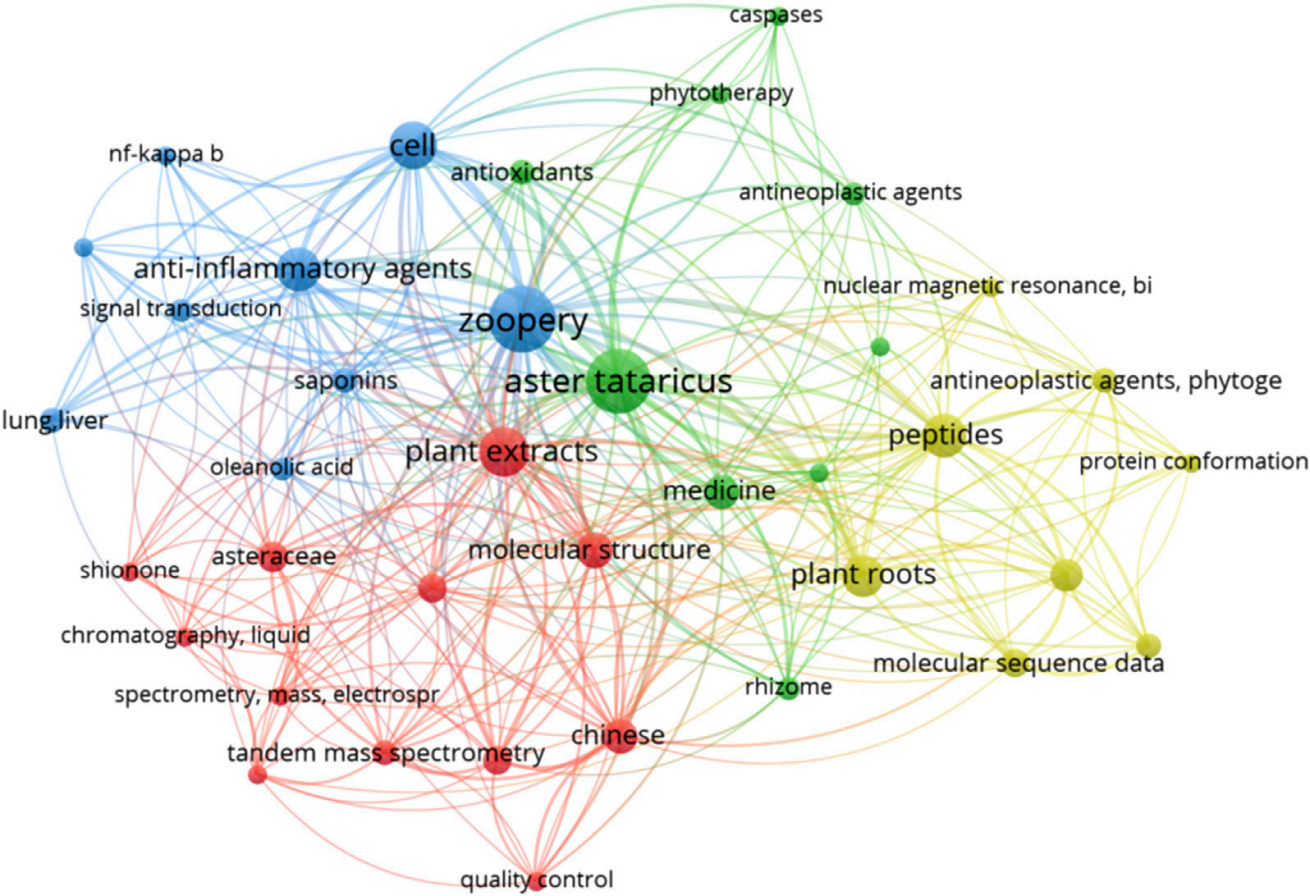
VOS viewer was used to visualise the keyword similarity across publications related to *A.tataricus*. The figure represents linear relationships between keywords, the larger the dot, the more frequently the word appears in the article. Many studies have explored the extraction of its chemical components, and most of *A.tataricus* research has been conducted using animal models.

According to the method described in the literature, VOS viewer software was used to visualize the similarity of all keywords in the literature reported before September 2024 (Figure 3). The figure shows that many studies have been conducted on the extraction of its chemical components, and most of the research on *A.tataricus* is concentrated on animal experiments.

At the same time, GBIF (www.gbif.org) and ArcGIS software were used to depict the distribution map of *A.tataricus*, biorender (https://www.biorender.com/) was used to draw the pharmacological mechanism map of *A.tataricus*, the flowchart templet from the PRISMA statement (https://www.prisma-statement.org/), and we utilised the iPlant (http://www.iplant.cn/) as a key resource to describe the botany and Photoshop was used to beautify the pictures.

## 3 Botany

**FIGURE 4:**
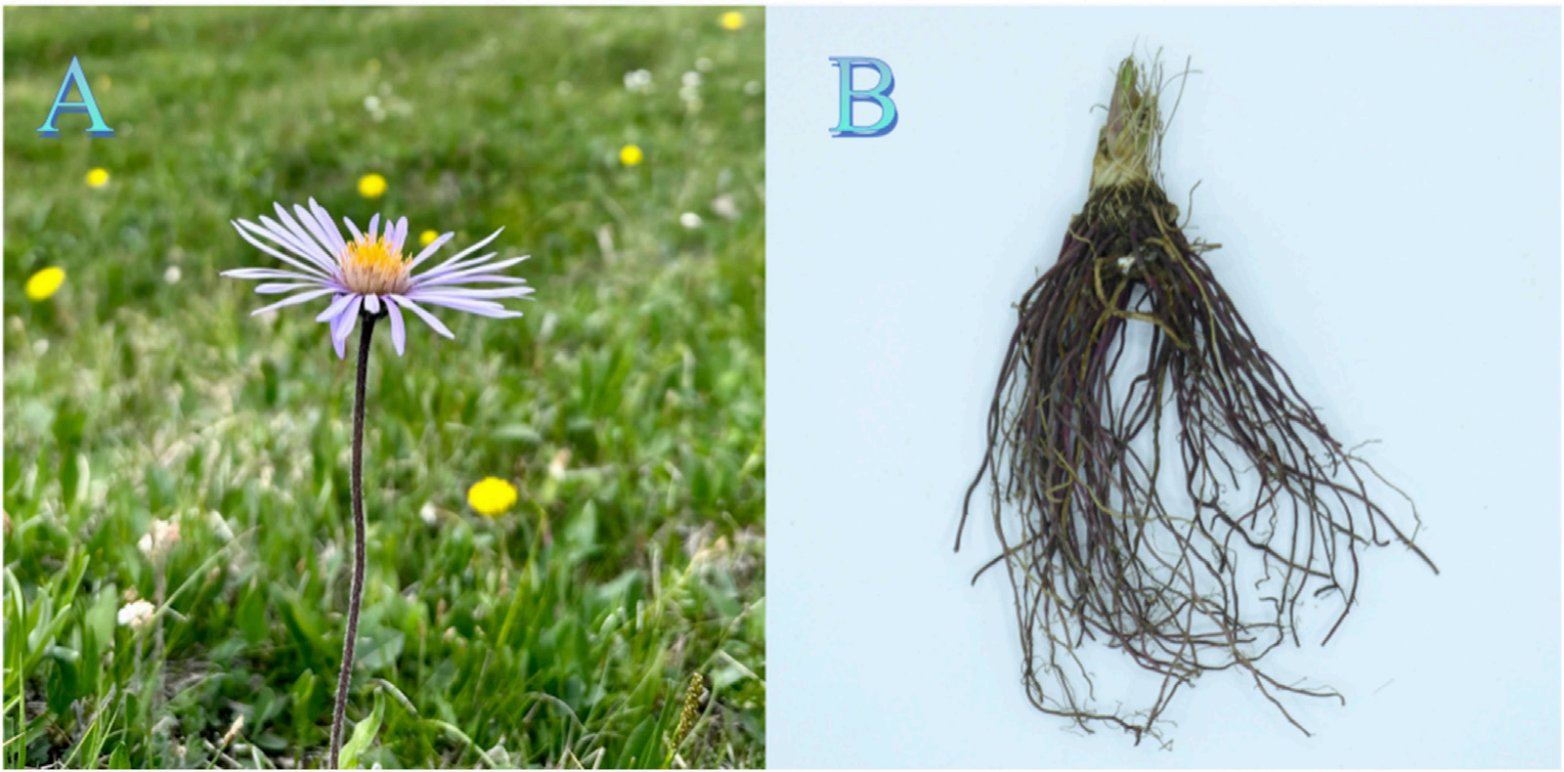
The morphology and medicinal parts of *A.tataricus.* **(A)** The aerial part of *A.tataricus* (photographed in the National Botanical Garden of China, Beijing). **(B)** The root and rhizome of *A.tataricus* (dried medicinal material).

During the Northern and Southern Dynasties of China, Hongjing Tao described the morphological characteristics of *A.tataricus* in *Ji Zhu of the Materia Medica*, his description—highlighting basal leaves, thick stem hairs, purple-colored tongue-shaped flowers, and soft roots—aligns with the current understanding of *A.tataricus* (Yu et al., 2023). After 1500 years of history, the shape of *A.tataricus* is now described as follows: The rhizome rises obliquely, the stem is upright and strong, reaching a height of 40–50 cm. With fibrous dead leaves at the base and adventitious roots, ribs, and grooves, covered with sparse, coarse hair and sparse leaves. The basal leaves wither during anthesis. These leaves are oblong or elliptic-spoon-shaped, the lower part tapers to grow into a stalk, and measure 20–50 cm in length and 3–13 cm in width. The leaf margin is either ornately lobed or shallowly serrated with small acute teeth. The leaves on the lower part of the stem are spatulate-oblong in shape, with their bases gradually tapering or abruptly narrowing into a broad-winged petiole; the outer margin is densely cleft except at the tip. The middle leaves are oblong to oblong-lanceolate, sessile, with entire margins or shallowly toothed. The upper lobe is narrow. The leaves are thickly papery in texture, with the upper surface being rough and the lower surface sparsely pubescent, featuring dense hairs along the veins. There are 6–10 pairs of lateral veins. The capitulum measures 2.5–4.5 cm in diameter and is mostly arranged in a compound corymbose cyme at the apex of the stem, with slender pedicels and linear bracteoles. The involucre is hemispherical, measuring 1–2.5 cm in diameter, with three layers of imbricate phyllaries that are linear to linear-lanceolate in shape, with apical or obtuse tips, densely puberulent, and with broadly membranous and reddish-purple margins. There are approximately 20 ligulate flowers, with the ligules being blue-purple. The achenes are obovate-oblong in shape, purplish-brown in color, and sparsely hirsute on the upper part. The corolla is simple, stained white or reddish, and composed primarily of strigose setae (Li H. et al., 2024) (Figure 4).

## 4 Artificial cultivation

Environmental factors such as soil composition, climate, and altitude have a significant impact on the efficacy of traditional Chinese medicine (TCM). Authentic medicinal herbs, which are synonymous with high-quality Chinese medicinal herbs, are selected through long-term clinical application of TCM, produced in a specific region, and have a high reputation. It has the characteristics of more stable quality and better efficacy (Huang et al., 2020). In China, *A.tataricus* is mainly produced in Hebei and Anhui provinces. It grows easily in low mountain shady slope wetlands, mountain tops, low mountain grassland, and marshland at an altitude of 400–2,000 m. Therefore, *A.tataricus* should be selected in loam soil with loose and high humus content or sandy loam. Through the review of previous studies, it was found that the bisexual and monosexual flowers of *A.tataricus* coexisted, self-pollination was the main method, and the seed setting rate was extremely low, which could not meet the seed demand of large-scale planting of *A.tataricus*. Therefore, most of the cultivation of *A.tataricus* uses roots and rhizomes as seedlings, so it is extremely important to establish a standardized system of high-quality seedlings (Yao et al., 2019). There are three main factors affecting the quality of seedlings: shoot hair number, shoot diameter, and shoot distance (Tian et al., 2012). The study found that (Song N. N. et al., 2024), the seedlings with strong, purplish red rhizomes, dense and short nodes, many dormant buds, white and tender broken bones, and no diseases and pests were selected. The young part at the lower end and the upper end of the reed head were removed, and the middle part was cut into small segments of 5–10 cm, with 2–3 bud eyes in each segment. Because *A.tataricus* leaves grow fastest from July to September, it is recommended to carry out spring planting (after the land is thawed, before the spring equinox term) and water 1–2 times before emergence. It likes to be moist, so it needs to be watered frequently to keep the soil surface moist, but it cannot be watered too much, which makes it easy to rot the roots. In addition to this, three weeding and fertilization procedures were performed during *A.tataricus* growth. The first time was after the whole seedling, the second time was about 7–10 cm in the seedling height, and the last time was before the plant was closed (Hu, 2014). Around the time of frost’s fall, that is, around the end of October, is the best time to harvest aster, and strong roots should be selected as seedlings at this time.

## 5 Nutrients and phytochemistry

**FIGURE 5:**
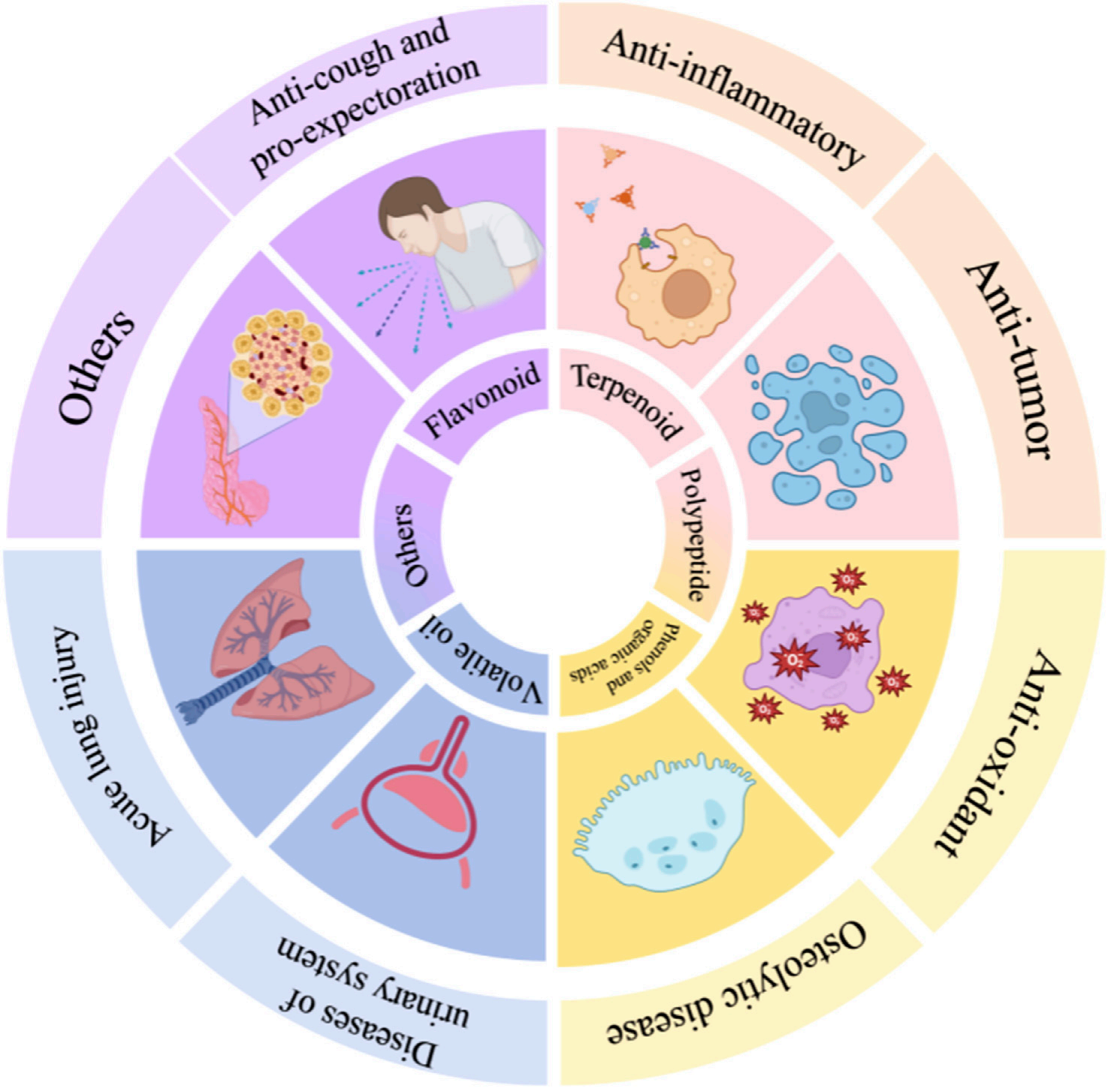
Key terpenoid compounds identified in *A.tataricus*.

Roots of *A.tataricus* (RA) contain 20 amino acids necessary for protein synthesis, including seven essential amino acids for humans: lysine, methionine, threonine, valine, leucine, isoleucine, phenylalanine, and histidine—the last one is particularly important for infants and young children. Among the free amino acids in RA, arginine and histidine have the highest mass fraction (Xi et al., 2003). Dietary arginine, in particular, supports wound healing, regulates endocrine function, and enhances immune activity (Morris, 2016) (Figure 5).

RA is also rich in trace elements, with high levels of Ca, Mg, and Fe, followed by Zn, Mn, Cu, and Mo, while Se is present in the lowest concentrations (Xi et al., 2003). A multitude of research indicates that the variety and density of inorganic elements in traditional Chinese medicinal practices play a pivotal role in the medicinal characteristics of their constituents (Ding, 2021; Hu, 2017; Li H. Y. et al., 2022; Chen W. et al., 2022). Using ICP-MS combined with stoichiometry, researchers compared inorganic elements of different medicinal parts of *A.tataricus*. Roots were found to have higher concentrations of U, V, Cr, Ga, and Ag, whereas rhizomes contained more Cu, Cd, Tl, and Zn (Li L. et al., 2023).

In 2018, Sun et al. (2018) identified and initially characterized 131 compounds in *A.tataricus*, including various bioactive compounds, such as terpenoids, polypeptides, flavonoids, phenols, organic acids, and volatile oils. Zhao et al. (2015) further isolated and determined phenolic acids, pentapeptides, and triterpene saponins in RA. Over the past 2 years, a new biflavonoid compound (Chen T. et al., 2022), 5 α-pyranone derivatives (Li X. et al., 2023), and five terpenoids (Li J. et al., 2024) have been discovered in *A.tataricus*. To date, approximately 200 chemical components have been successfully isolated and confirmed from *A.tataricus*, among which terpenoids are the most abundant, accounting for approximately 40% of the total components (Fan et al., 2024).

### 5.1 Terpenoids

**FIGURE 6:**
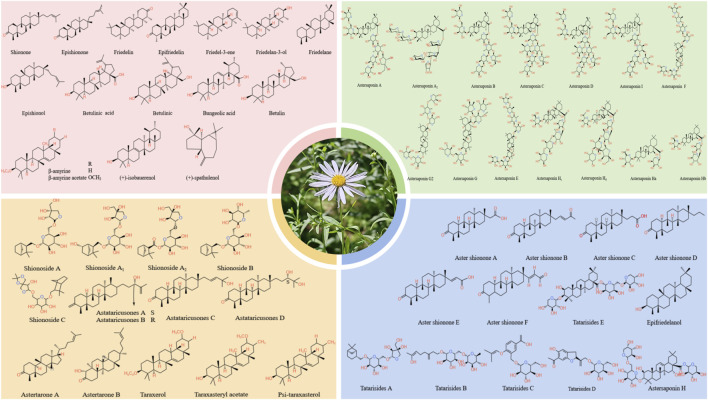
The primary flavonoids in *A.tataricus*.

Terpenoids are the primary active substances in *A.tataricus.* To date, 66 terpenoids have been identified in *A.tataricus*, and their chemical structures are listed in Table 1 and Figure 6. Studies have shown that the predominant component in *A.tataricus* is shionone-type triterpene compounds. As the first isolated and structurally determined compound in *A.tataricus*, shionone is a unique component of this plant, characterised by a complete six-membered four-ring skeleton and a unique structure of 3-oxo-4-monomethyl. Ninety percent of shionone is synthesised in a complex pathway mediated by a single enzyme, shionone synthase (Sawai et al., 2011). As a quality marker, *ChP* stipulates that authentic *A.tataricus* must contain no less than 0.10% shionone (Jaiswal and Lee, 2023). Studies have shown that (Zhang et al., 2022) the content of shionone in fresh medicinal materials is approximately three times that of traditional Chinese medicine pieces, and the content varies greatly in different medicinal materials. The content in roots is about 100 times that of stems and flowers. In roots, the content of shionone also varies. The content of shionone in roots is higher than that in main roots and rhizome. At the same time, some studies have shown that processing methods (Yang et al., 2021), producing area (Wu et al., 2003), and harvest season also affect the content of shionone.

**TABLE 1:** Names, molecular formulas, harvesting parts, extraction reagents and literature sources of terpenoids in *A.tataricus.*

No.	Components	Molecular formula	Parts	Extracting solvent	References
1	Aster saponin A	C_68_H_110_O_33_	Roots	ODS	Yu et al. (2015a)
2	Aster saponin A_2_	C_62_H_100_O_29_	Underground parts	methanol	Su et al. (2019b)
3	Aster saponin B	C_62_H_100_O_29_	Roots	ODS	Nagao et al. (1989)
4	Aster saponin C	C_74_H_120_O_37_	Roots	MeOH	Nagao et al. (1989)
5	Aster saponin D	C_73_H_188_O_36_	Roots	MeOH	Nagao et al. (1989)
6	Aster saponin E	C_63_H_109_O_29_	Roots	MeOH	Stonik et al. (2020)
7	Aster saponin F	C_63_H_108_O_28_	Roots	MeOH	Stonik et al. (2020)
8	Aster saponin G	C_57_H_92_O_26_	Underground parts	methanol	Su et al. (2019b)
9	Aster saponin H_a_	C_38_H_58_O_13_	Aboveground parts	MeOH	Su et al. (2019b)
10	Aster saponin H_b_	C_42_H_66_O_13_	Aboveground parts	MeOH	Tanaka et al. (1990)
11	Aster saponin H_c_	C_58_H_92_O_25_	Aboveground parts	MeOH	Tanaka et al. (1990)
12	Aster saponin Hd	C64H102O26	Aboveground parts	MeOH	Tanaka et al. (1990)
13	Foetidissimo-side A	C_56_H_90_O_18_	Aboveground parts	MeOH	Tanaka et al. (1990)
14	Shionone	C_30_H_50_O	Roots and rhizomes	95%EtOH	Jaiswal and Lee (2023)
15	EpiShionone	C_30_H_50_O	Roots and rhizomes	*n*-hexane	Sawai et al. (2011)
16	Friedelin	C_30_H_50_O	Roots and rhizomes	*n*-hexane	Sawai et al. (2011)
17	Epifriedelin	C_30_H_50_O	Roots and rhizomes	MeOH	Yin et al. (2016)
18	Friedel-3-ene	C_30_H_50_O	Roots and rhizomes	MeOH	Lanzotti (2005)
19	Shionoside A	C_21_H_36_O_10_	Roots and rhizomes	MeOH	Su et al. (2019a)
20	Shionoside A_1_	C_21_H_36_O_10_	Roots and rhizomes	MeOH	Su et al. (2019b)
21	Shionoside A_2_	C_21_H_36_O_10_	Roots and rhizomes	MeOH	Su et al. (2019a)
22	Shionoside B	C_22_H_38_O_10_	Roots and rhizomes	MeOH	Tian et al. (2021)
23	Shionoside C	C_24_H_40_O_10_	Roots	70% ethanol	Cheng and Shao (1994)
24	Tatarisides A	C_21_H_36_O_10_	Roots and rhizomes	70% EtOH	Li et al. (2024b)
25	Tatarisides B	C_22_H_38_O_10_	Roots and rhizomes	70% EtOH	Li et al. (2024b)
26	Tatarisides C	C_19_H_26_O_8_	Roots and rhizomes	70% EtOH	Li et al. (2024b)
27	Tatarisides D	C_19_H_24_O_9_	Roots and rhizomes	70% EtOH	Li et al. (2024b)
28	Tatarisides E	C_46_H_74_O_9_	Roots and rhizomes	70% EtOH	Li et al. (2024b)
29	Astataricusones A	C_30_H_50_O_2_	Roots and rhizomes	MeOH	Zhou et al. (2013)
30	Astataricusones B	C_30_H_50_O_2_	Roots and rhizomes	MeOH	Zhou et al. (2013)
31	Astataricusones C	C_30_H_50_O_2_	Roots and rhizomes	MeOH	Zhou et al. (2013)
32	Astataricusones D	C_30_H_52_O_3_	Roots and rhizomes	MeOH	Zhou et al. (2013)
33	Astataricusol A	C_30_H_52_O_2_	Roots and rhizomes	MeOH	Zhou et al. (2013)
34	(+)-isobauerenol	C_30_H_50_O	Roots and rhizomes	MeOH	Su et al. (2019a)
35	(4α)-17-(acetyloxy)kauran-18-oic acid	C_21_H_34_O_3_	Roots and rhizomes	MeOH	Su et al. (2019a)
36	(+)-spathulenol	C_15_H_24_O	Roots and rhizomes	MeOH	Su et al. (2019a)
37	Astertarone A	C_30_H_50_O	Roots	MeOH	Jin et al. (2018)
38	Astertarone B	C_31_H_52_O_2_	Roots	MeOH	Akihisa et al. (1999)
39	β-amyrine	C_30_H_50_O	Roots and rhizomes	Methanol	Sun et al. (2018)
40	β-amyrine acetate	C_32_H_52_O_2_	Roots and rhizomes	Methanol	Sun et al. (2018)
41	Taraxerol	C_30_H_50_O	Roots and rhizomes	Methanol	Sun et al. (2018)
42	Taraxasteryl acetate	C_32_H_52_O_2_	Roots and rhizomes	Methanol	Sun et al. (2018)
43	psi-taraxasterol	C_30_H_50_O	Roots and rhizomes	Methanol	Sun et al. (2018)
44	Friedelan-3-ol	C_30_H_52_O	Roots	MeOH	Lanzotti (2005)
45	23-Hydroxybetulinic acid	C_30_H_48_O_4_	Roots and rhizomes	Methanol	Sun et al. (2018)
46	Betulinic acid	C_30_H_48_O_3_	Roots and rhizomes	Methanol	Sun et al. (2018)
47	Betulinic	C_30_H_52_O_2_	Roots and rhizomes	Methanol	Sun et al. (2018)
48	Shion-22-methoxy-20 (21)-en-3-one	C_30_H_52_O_2_	Roots and rhizomes	95%EtOH	Zhou et al. (2010)
49	Shion-22 (30)-en-3,21-dione	C_30_H_48_O_2_	Roots and rhizomes	95%EtOH	Zhou et al. (2010)
50	shion-22-methoxy-20 (21)-en-3β-ol	C_31_H_54_O_2_	Roots and rhizomes	95%EtOH	Zhou et al. (2010)
51	Stigmasterol-β-D-glucoside	C_35_H_58_O_6_	Roots and rhizomes	MeOH	Su et al. (2019b)
52	hept-2-yl)methyl-O-β-D-glucopyranoside	C_21_H_36_O_10_	Roots and rhizomes	MeOH	Luo et al. (2024)
53	Epishionol	C_30_H_52_O	Roots and rhizomes	95% EtOH	Zhou (2010)
54	Friedelane	C_30_H_52_	Roots and rhizomes	95% EtOH	Zhou (2010)
55	Epifriedelanol	C_30_H_52_O	Roots and rhizomes	95% EtOH	Zhou (2010)
56	24-Ethyl-5a-cholesta-7,22(E)-dien-3-one	C_34_H_56_O	Roots and rhizomes	95% EtOH	Zhou (2010)
57	α-Spinach sterols	C_29_H_48_O	Roots and rhizomes	Ethyl acetate	Ye (2007)
58	Oleanolic acid	C_30_H_48_O_3_	Roots and rhizomes	Methanol	Sun et al. (2018)
59	2,3,24-Trihydroxyolean-12-en-28-oic acid	C_30_H_48_O_5_	Roots and rhizomes	Methanol	Sun et al. (2018)
60	Aster saponin G_2_	C_57_H_92_O_25_	Underground parts	Methanol	Su et al. (2019b)
61	Aster saponin H	C_46_H_74_O_18_	Underground parts	Methanol	Su et al. (2019b)
62	Aster saponin C_2_	C_68_H_110_O_33_	Underground parts	Methanol	Su et al. (2019b)
63	3-O-α-L-arabinopyranosyl-(1 → 6)-β-D-trihydroxyolean -12-en-28-oic acid	C_41_H_66_O_14_	Underground parts	Methanol	Su et al. (2019b)
64	Aster Shionone D	C_27_H_44_O_3_	Roots and rhizomes	MeOH	Zhou et al. (2014)
65	Aster Shionone E	C_27_H_46_O	Roots and rhizomes	MeOH	Zhou et al. (2014)
66	Aster Shionone F	C_27_H_42_O_3_	Roots and rhizomes	MeOH	Zhou et al. (2014)

### 5.2 Flavonoids

**FIGURE 7:**
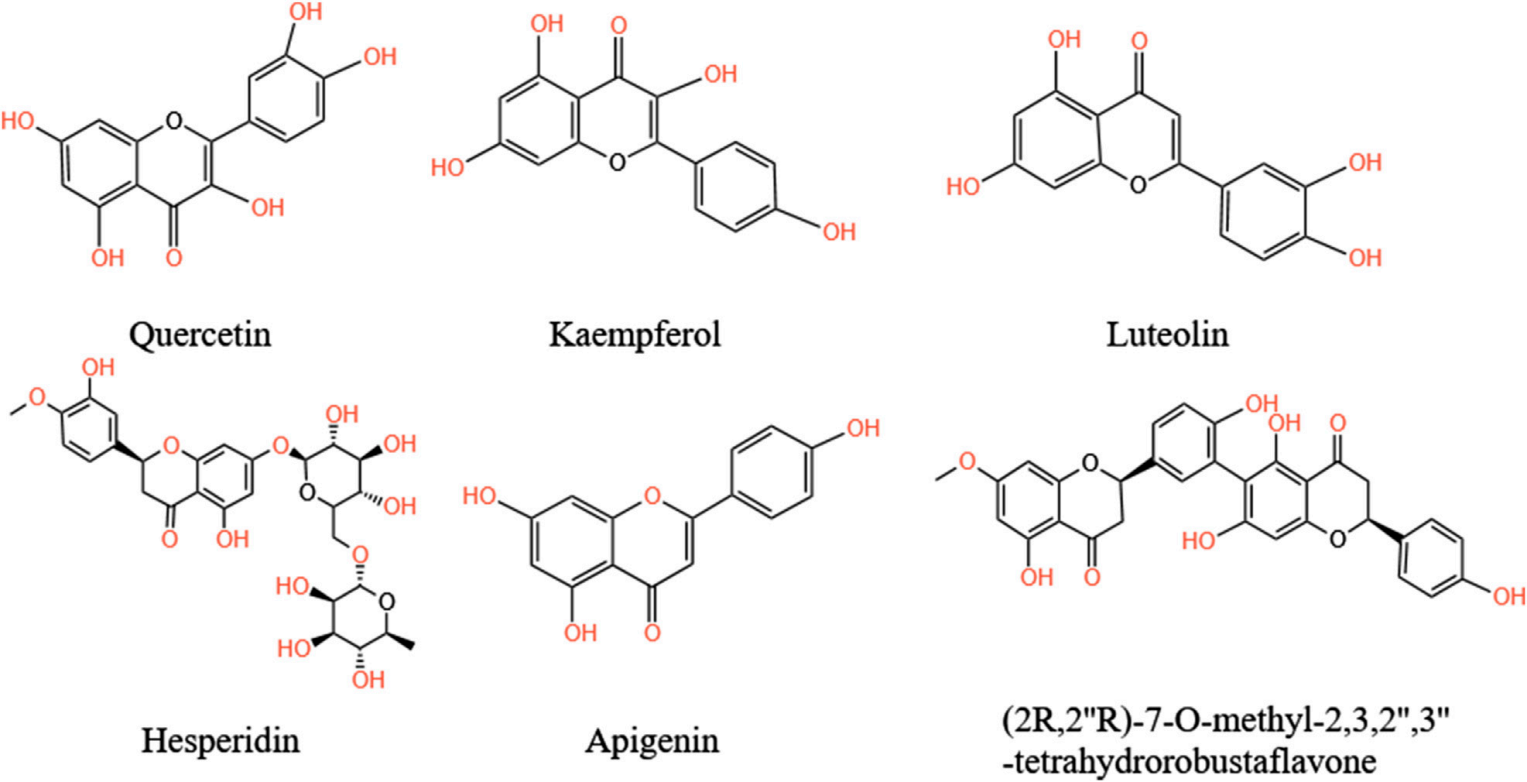
Polypeptide constituents of *A.tataricus.*

Flavonoids, widely found in plants, regulate their physiological functions. They are the largest class of secondary metabolites in plants and exhibit potent pharmacological activities (Liu et al., 2021). Jia et al. (2023) identified more than 80 flavonoid metabolites in *A.tataricus* through multiple omics methods. The metabolites included 31 flavonols, 29 flavonoids, six isoflavones, five dihydroflavonoids, five anthocyanins, two flavonoid carbon glycosides, and one each of dihydroflavonol and flavanol. In recent years, from the MeOH extract of *A.tataricus*, a novel biflavonoid compound of the C-3′-C-6″ variety, termed (2R,2″R)-7-O-methyl-2,3,2″,3″-tetrahydrorobustaflavone, was extracted (Chen T. et al., 2022) (Figure 7). At present, the determination of flavonoid content in *A.tataricus* mainly focuses on quercetin and kaempferol, which mainly accumulate in the cortex and pith (Guo et al., 2016). After measuring the quercetin content in different parts of *A.tataricus* in different areas, it was found that the quercetin content in the roots and rhizomes is considerably higher than that in the flowers (Song et al., 2009).

### 5.3 Polypeptides

**FIGURE 8:**
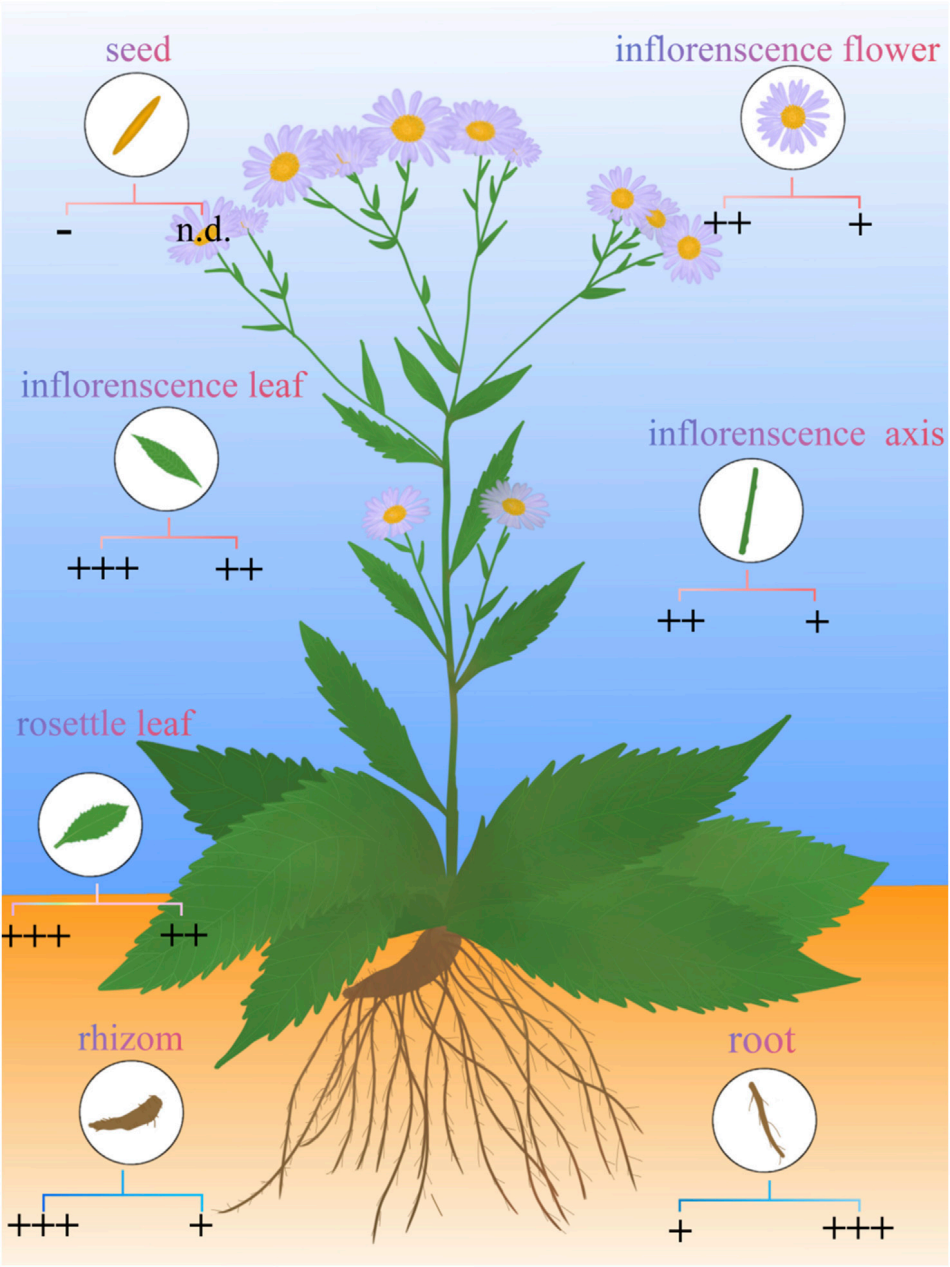
Distribution of shionone and astins in *A.tataricus* (represented in circular symbols in different tissues). The concentrations of shionone and astins are represented in sign below, the left is the concentration of astins, the right is the concentration of shionone, astins concentration:+: <5 × 10^11^, ++: 5 × 10^11^–5 × 10^12^, +++: >5 × 10^12^, N.D.: Unknown concentration; shionone concentration: +: <0.15%, ++: 0.15%–0.3%, +++: >0.3%. Data source: (Chen et al., 2020; Gao et al., 2003; Zhang et al., 2022; Zhang et al., 2021; Schafhauser et al., 2019).

**FIGURE 9:**
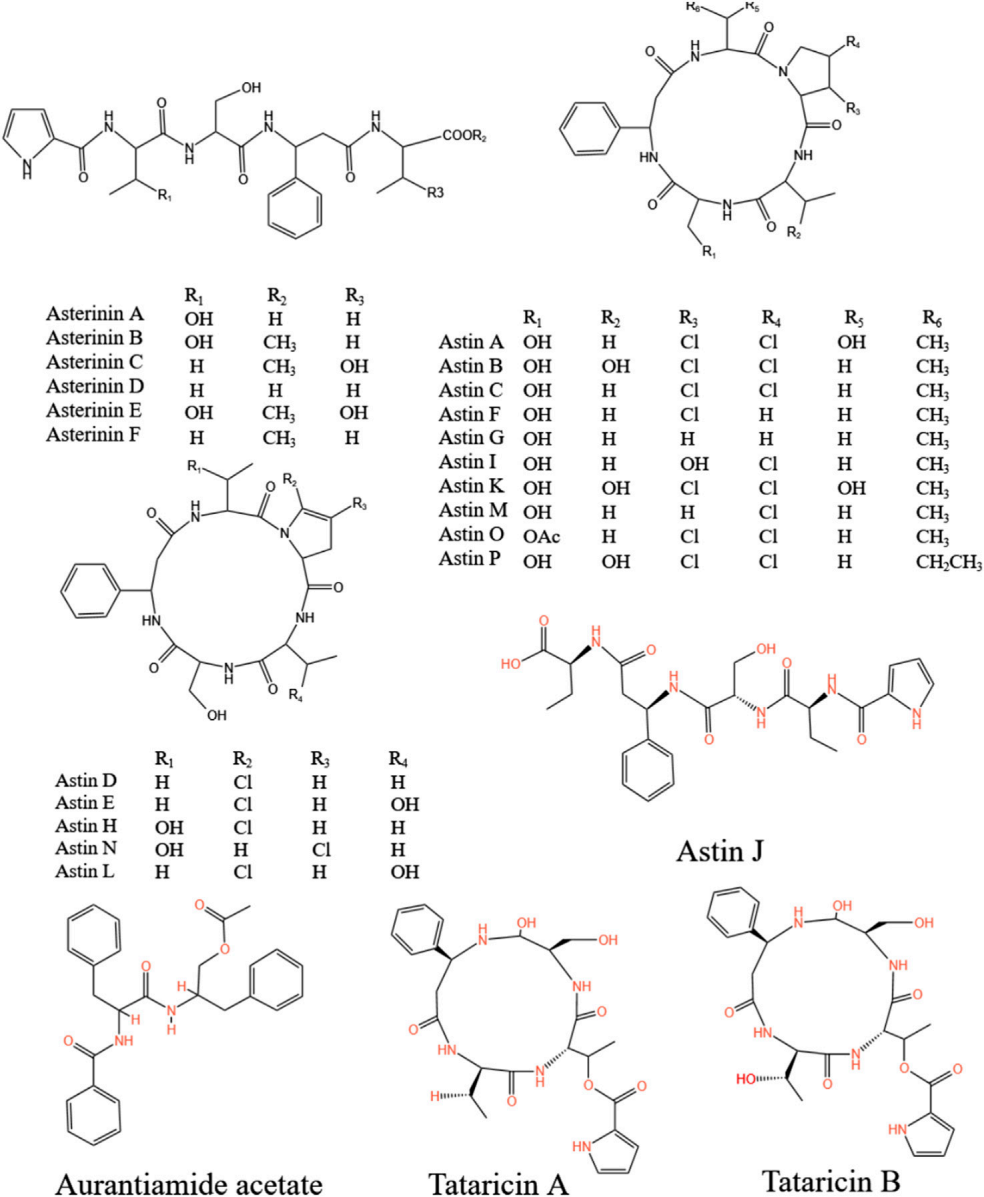
Major phenolic and organic acid compounds in *A.tataricus*.

Peptides also stand out as distinct elements in *A.tataricus* (Figure 8). Currently, 28 peptides have been isolated from *A.tataricus*, including halogenated cyclic polypeptides (astins A-P, and tataricins A-B) and linear polypeptides (asterinins A-F, and aurantiamide acetate) (Zhao et al., 2015). Studies found that (Rossi et al., 2004) astins are only found in the root of *A.tataricus*, and their conformational flexibility is closely linked to the biological activity of their family members. In particular, cyclic astin family members, in the form of cyclic peptides, inhibit the growth of anti-tumor cell lines, whereas non-cyclic astins structures do not exhibit similar anti-tumor activity. Cyclic pentapeptides in the astin family are distinguished by having four amino acids that are not proteins, of which dichloroproline and isothreonine are the most prominent characteristic residues (Cozzolino et al., 2005). In 1998, Lu et al. (Lu et al., 1998) discovered and isolated a dipeptide compound aurantiamide acetate from the petroleum ether extracts of the roots and rhizomes of *A.tataricus*. This component exhibited inhibitory activity against superoxide radical formation. In 2013, Xu et al. (2013) identified eight new polypeptide compounds from *A.tataricus*, including two new cyclic tetrapeptides, tataricins A and tataricins B. These two compounds have unique cyclic peptide skeletons and ^△2,4^Pro side chains. Additionally, six novel chlorinated cyclopentanone peptides were isolated, including Astin P, a cyclic pentapeptide with the sequence (L-Pro(Cl2)1-L-allo-Thr2-LSer3-L-b-Phe4-L-ava5) (Figure 9). A study by Schafhauser et al. (2019) pointed out that astins originate from the endophytic fungus *Cyanodermela astris*, which is associated with *A.tataricus*. These compounds are products of the NRPS biosynthetic pathway. They exist not only in roots and rhizomes but are also abundant in leaves and inflorescences.

### 5.4 Phenols and organic acids

**FIGURE 10:**
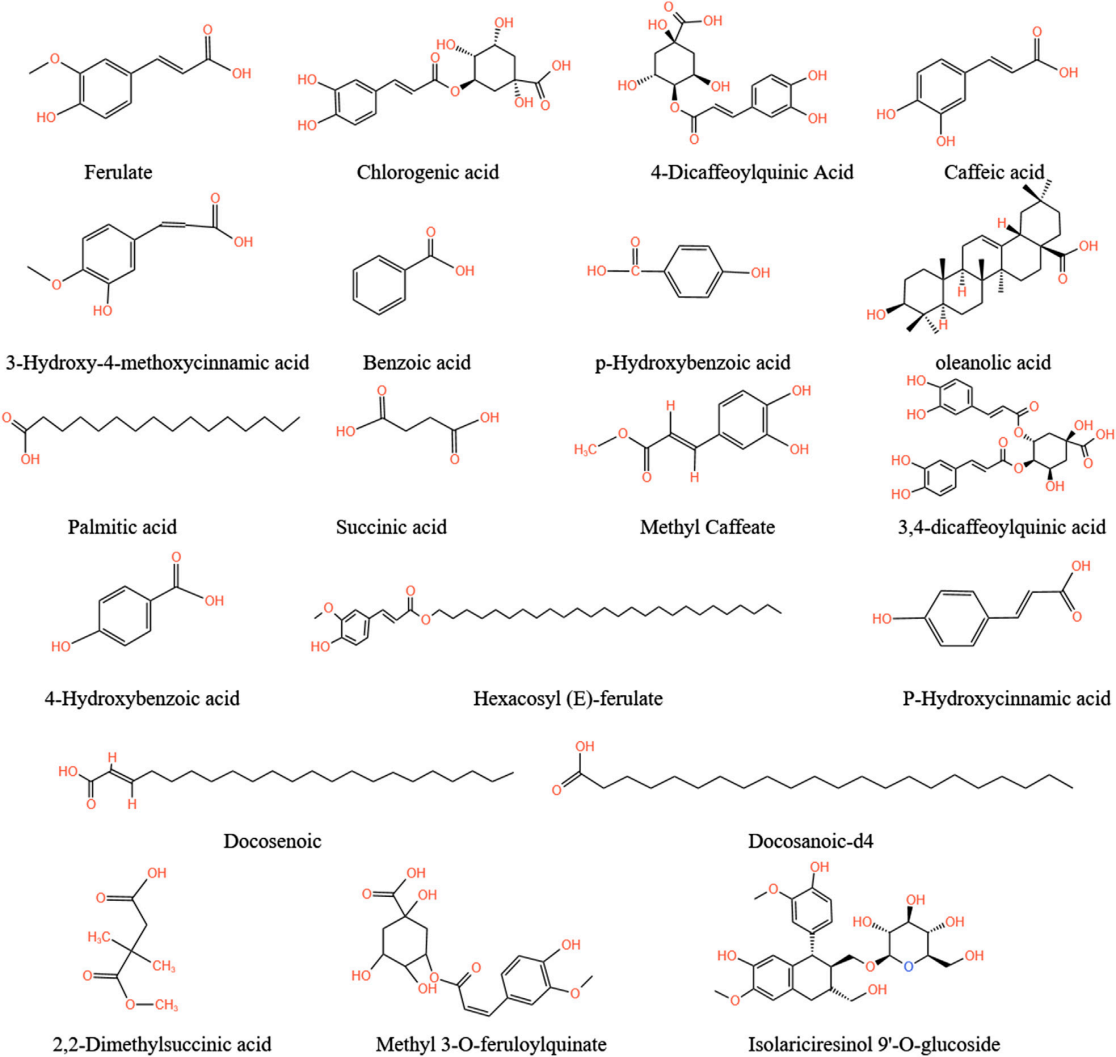
Structures of selected volatile oil components in *A.tataricus.*

The organic acids in the medicinal parts of *A.tataricus* are primarily aromatic acids (Sun et al., 2018). An evaluation of three chlorogenic acid contents in different underground parts of *A.tataricus* revealed that the rhizome had the highest chlorogenic acid content, followed by the parent root. In contrast, the root had the lowest content (Wang et al., 2020a). Tian et al. (2008) simultaneously determined three chemical components and found that *A.tataricus* contains a high content of ferulic acid. In addition to chlorogenic acid and ferulic acid, a variety of organic acid components (including polyphenols) are present in *A.tataricus*, including cryptochlorogenic acid, caffeic acid, isoferulic acid, benzoic acid, para-hydroxybenzoic acid, oleanolic acid, palmitic acid, succinic acid, caffeic acid methyl ester, 3,4-dicaffeoylquinic acid, 4-hydroxybenzoic acid, dicetyl ester, cetyl p-hydroxycinnamate, docosanoic acid, behenic acid, 2, 2-dimethylsuccinic acid (Jin et al., 2008) and the phenolic compounds 3-O-feruloylquinate methyl ester and (+)-isolaricidin- 9-β-D-glucopyranoside (Gao et al., 1994) (Figure 10).

### 5.5 Volatile oil

**FIGURE 11:**
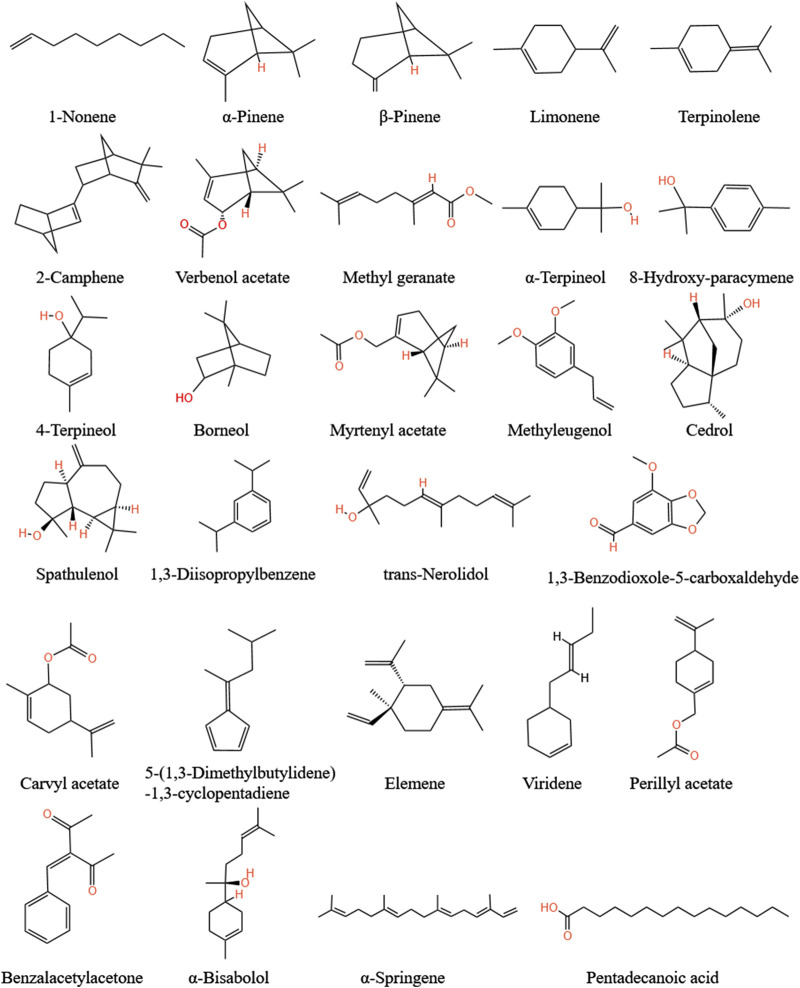
Structural formulae of selected compounds in *A.tataricus*.

Yang et al. (2008) identified seven volatile oil components and isolated 1-acetoxy-2-ene(E)-4,6-decandiyne. Zhang J. L. et al. (2012) analyzed and found that the content of 1-acetoxy-2-ene(E)-4,6-decandiyne is the most abundant volatile oil i in *A.tataricus*, followed by 32 volatile oil components, such as 5-(1,3-dimethylbutylidene)-1,3- cyclopentadiene, γ-elemene, m-diisopropyl, α-springene, τ-cadinol, and β-pinene (Figure 11).

### 5.6 Other compounds

**FIGURE 12:**
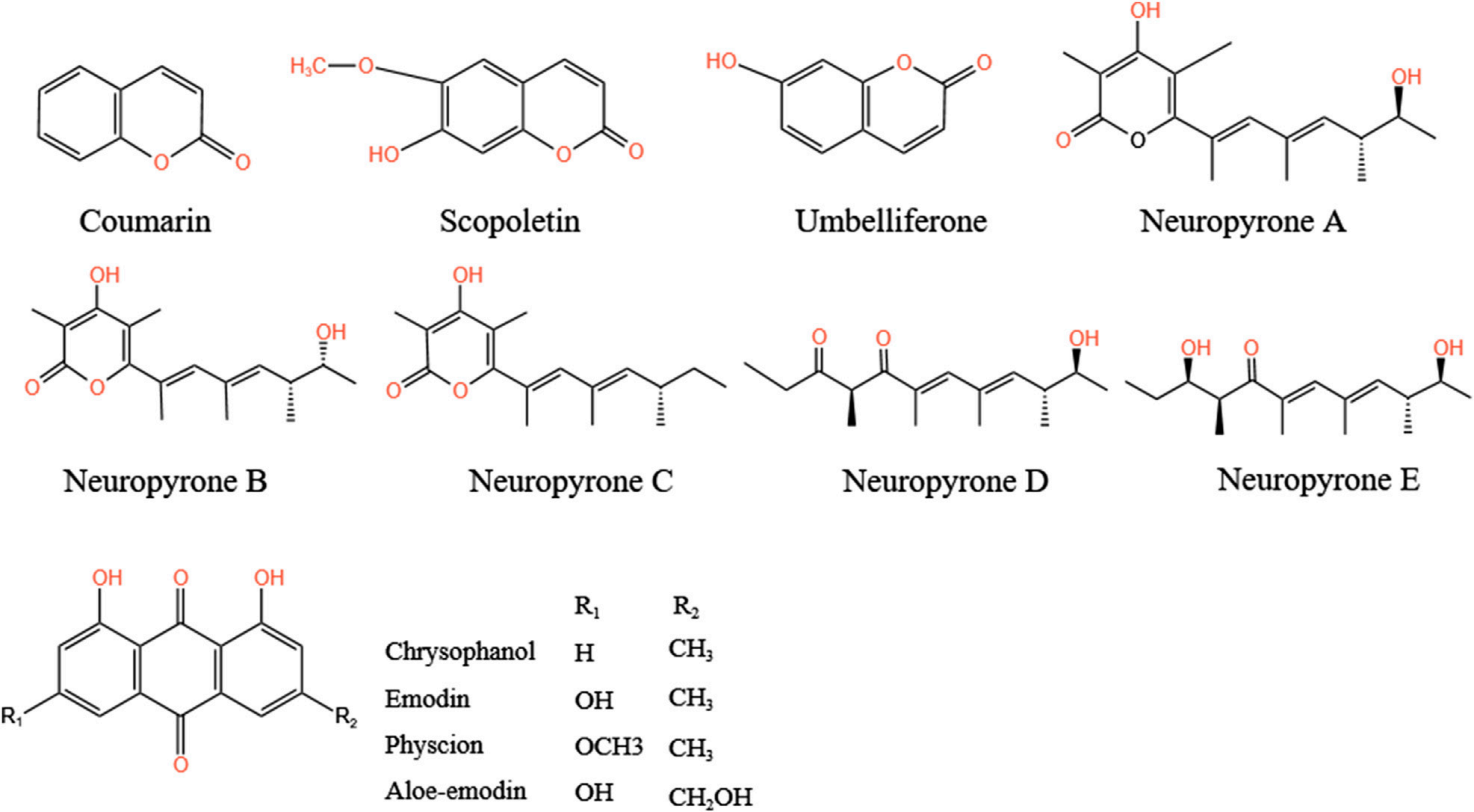
Chemical constituents and pharmacological actions of *A.tataricus.*

In addition to the compounds mentioned above, *A.tataricus* also contains polysaccharides, coumarins, sterols, and anthraquinone derivatives (Fan et al., 2024). Two alkanes, n-octadecane and n-tricanol, were also separated from the methanol extract (Ye, 2007). Acetylene and other substances have been isolated from the ethanol extract of *A.tataricus* (ATEE) (Zhou, 2010). Among them, coumarin compounds include coumarins, scopoletin, and umbellitone (Cai et al., 2023). Li X. et al. (2023) identified five unidentified α-pyranone derivatives, namely neuropyrones A–E, from the endophytic fungus Neurosporadictyophora WZ-497. Du et al. (2014) successfully purified a homogeneous polysaccharide (ATP-II) consisting of glucose, galactose, mannose, rhamnose, and arabinose (2.1: 5.2: 2.1: 1.0: 1.2) from *A.tataricus*. Zhang Y. et al. (2012) isolated A water-soluble polysaccharide (WATP) composed of galactose, glucose, fucose, rhamnose, arabinose, and mannose (2.1: 1.3: 0.9: 0.5: 0.3: 0.6) from *A.tataricus*. Wang et al. (2003) isolated anthraquinones in *A.tataricus* from the roots and rhizome extracted by ethyl acetate, including emodin, chrysophanol, aloe-emodin, and physcion methyl ether (Figure 12), whereas Ng et al. (2003) isolated 1,7-dihydroxy-6-methyl-anthraquinone, and the bisanthraquinone skyrin from *A.tataricus.* Skyrin was identified as the main colorant of its endophytic fungus *Cyanodermela astris* (Jahn et al., 2017). In addition, amide compounds such as [N-(N-benzoyl-L-phenylalanoyl)-O-acetyl-L-phenylalanine] (Zou et al., 1999), and furan compounds, including 11-hydroxy-10,11-dihydro-euparin (Jin et al., 2008) are present in *A.tataricus*. At present, there are few studies on steroids in *A.tataricus* and only three sterols have been found, isolated, and identified, namely stigmasterol, β-sitosterol, and daucosterol. In addition, Tang et al. (2006) extracted 0.1506% lipid-soluble total alkaloids from *A.tataricus*.

## 6 Ethnopharmacology

### 6.1 Traditional application

*A.tataricus* is distributed across China, Korea, Japan, the northern United States, and eastern Siberia in Russia. Over millennia, *A.tataricus’s* roots, stems, leaves, and whole plants have served diverse medicinal roles in traditional folk medicine across southeast Asia. In China, *A.tataricus* is known as “*Zi Wan*” or “*Huan Hun Cao*”. *A.tataricus* was first recorded in *Shuo Wen* under the alias “*Zi Wan*” (茈菀). Table 2 records the herbal research of *A.tataricus*. *Shennong Materia Medica* used the name “*Zi Wan*” (紫菀), which has been carried over subsequent generations. *A.tataricus* is an important drug for clearing and moistening the lungs. Its pharmacological effect was first recorded in *Shennong’s Herbal Classic* (Li, 1800). The whole herb is mainly used for treating coughs caused by the upward flow of qi, cold-heat condensation in the chest, poison removal, paralysis, and soothing the five internal organs. The parts used are mainly dry roots and stems. The *Supplementary Records of Famous Physicians* (Tao, 2013) state that “*A.tataricus* treats symptoms such as coughing up pus and blood, asthma, palpitations, fatigue, or lesions in the five internal organs. It is also used to treat weakness, address deficiencies, and childhood epilepsy. The *Xinxiu Materia Medica* of the Tang Dynasty integrates the descriptions of the sexual and taste effects of *A.tataricus* in the *Shennong Materia Medica Classic* and *Supplementary Records of Famous Physicians. A.tataricus* is primarily used to treat lung-related diseases. During the Tang and Song Dynasties, *Rihuazi Materia Medica* (Ri and Shang, 1983) compared with its predecessors, it has introduced additional therapeutic uses, including quenching thirst, moisturising skin, and replenishing bone marrow. In terms of clinical applications, *Qianjin Fang* (Sun, 2019) recorded a prescription for treating women with oliguria and hematuria. During the Jin and Yuan Dynasties, Xueyue Jia noted in *Medicine Huayi* that (Jia, 2015) *A.tataricus* was mainly used for chronic coughing, expectoration, coughing up blood, lung weakness, and diabetes in the lung system. Additional indications for use in the liver and kidney meridians included insufficient fatigue and heat in the liver meridian, heat accumulation and stagnation of qi, vomiting blood and bleeding in the stool, dry stools, and short and red urine in the kidney system. Modern texts, such as the *Chinese Materia Medica* (Administration C. F. A. D, 1999) described the effects of *A.tataricus* as “moistening the lungs and lowering the qi, alleviating phlegm and easing cough.” It is primarily used for lung deficiency, chronic cough, lung weakness, and coughing up pus or blood, aligning with its historical applications. The 2020 edition of the ChP reiterates these uses. In conclusion, *A.tataricus* helps relieve lung qi, pass body fluid, calm adverse-rising energy, relieve cough, resolve phlegm, and smooth secretions (Yu et al., 2023).

**TABLE 2:** *A.tataricus* related formulas and their chemical components and biological activity effects.

Formula name	Source	Ingredients	Efficacy
Ziwan Bemu Wan	Prescriptions for Universal Relief	A.tataricus 2.5 g, thunberbg fritillary bulb 2.5 g, Pinellia ternata 2.5 g, Mori Cortex 2.5 g, Schisandrae Chinensis Fructus 2.5 g, Belamcandae Rhizoma 2.5 g, Stemonae Radix 2.5 g, Farfarae flos 30 g, Zanthoxyli Pericapium 30 g, Zingiberis Rhizoma 30 g, etc.	Expectorant and cough-relieving, lung-moistening and asthma-relieving, used for cough, phlegm abundance, and wheezing
Zhisou San	Medical Insight	Platycodonis Radix 10 g, Schizonepetae Herba 10 g, A.tataricus 10 g, Stemonae Radix 10 g, Cynanchi Stauntonii Rhizoma et Radix 10 g, Glycyrrhizae Radix et Rhizoma 4 g, Citri Reticulatae Pericarpium 5 g	Expectorant and cough-relieving, lung-moistening and asthma-relieving, used for cough due to lung invasion by wind evil, and is commonly used for modern upper respiratory tract infections and the like
Baiqian Decoction	Beiji Qianjin Yao Fang	Cynanchi Stauntonii Rhizoma et Radix, A.tataricus, Pinelliae Rhizoma, Cirsii Japonici Herba each 15 g	Expectorant and cough-relieving, used for cough with upward - moving qi, body edema, shortness of breath, abdominal distention, etc
Fufang Qingfei Zhige Tang	Journal of Traditional Chinese Medicine	Gypsum fibrosum 30 g, Anemarrhenae Rhizoma 15 g, Glycyrrhizae Radix et Rhizoma 9 g, Rehmanniae Radix 15 g, Sanguisorbae Radix 15 g, Eriobotryae Folium 12 g, A.tataricus 15 g, etc.	Liver-quieting and lung-clearing, blood-stanching and collateral-stabilizing, used for lung dryness-heat, liver fire exuberance, lung network damage, and blood flowing recklessly

In Japanese Kampo medicine, *A.tataricus* is commonly used in compound preparations, mainly for treating respiratory diseases, such as for expectorant and cough-stopping purposes, for moistening the lungs, and for exerting antibacterial and anti-inflammatory effects (Commission J. P, 2023). In traditional Korean medicine, it is also often used to treat coughs and asthma (Kang et al., 2021). However, in China, its roots and rhizomes are primarily used for medicinal purposes (Wang et al., 2020b), whereas in South Korea, its flowers are used (Chen et al., 2012). So, many ancient documents from Chinese traditional medicine, Korean medicine, and Japanese traditional medicine have recorded the effects of *A.tataricus* on relieving cough and phlegm (Su et al., 2019a). Despite varying cultural differences, the historical use of *A.tataricus* reveals a consistent global trend. Hence, the establishment of a thorough drug quality standard system becomes imperative to effectively support quality control and the advancement of new drug development.

### 6.2 Processing

The practice of processing holds significant importance in the realm of traditional Chinese medicine, as it can reduce the toxicity of herbs and improve their efficacy. This is true of *A.tataricus* as well. When detecting the chemical composition of six *A.tataricus* processed slices, namely raw, honey-fried, stir-fried, vinegar-fried, wine-fried, and steamed, it was found that stir-fried *A.tataricus* had the highest content of shionone and flavonoids, while raw *A.tataricus* had the lowest content of shionone (Fan et al., 2018). After honey-fried, the shionone content in *A.tataricus* significantly increases, while flavonoid levels drop. Its expectorant and cough-relieving effects become more pronounced. In contrast, in the stir-fried product, only shionone shows a marked rise, with flavonoid content remaining largely unchanged (Wang X. et al., 2023; Wu et al., 2006; Xiu et al., 2006). Shizhen Li pointed out in *The Compendium of Materia Medica* that the spicy nature of *A.tataricus* can strongly affect the body but may also damage lung yin (Li et al., 2022b). Hence, it is used in conjunction with other medicinal materials to treat wind-cold cough, asthma, fatigue-induced coughing, vomiting, pus formation, bleeding, and other symptoms. For example, in *Zhang’s Yi Tong*, *A.tataricus* is combined with drugs such as Ejiao, *Fritillaria*, *Ophiopogon japonicus* L. f. (*O.japonicus*), and *Schisandra chinensis* Turcz. to address deficiencies, fatigue, lung weakness, and hemoptysis. It is decocted with *Glycyrrhiza uralensis* Fisch, *Asparagus cochinchinensi*, *Platycodon grandiflorus* (*P.grandiflorus*), honey, *Amygdalus Communis* Vas, and *Mori Cortex* to treat coughing and threatened abortion (Yu et al., 2023). *A.tataricus* is often paired with *Tussilago farfara* L. (*T.farfara*), *Stemona japonica*, and *Ephedrae Herba* as a standard method for treating pulmonary diseases. When combined with *P. grandiflorus*, Poria, and *O. japonicus*, it enhances the effects of dispersing lung qi and promoting water metabolism. Additionally, when used with *Coicis Semen*, *P. grandiflorus*, and *Amygdalus communis* Vas., it further strengthens the actions of dispersing lung qi and relaxing the bowel (Tian et al., 2021). One of the most common prescriptions in China, *Zhishou powder* (止嗽粉), uses *A.tataricus* as the primary ingredient (monarch medicine) and is extensively employed in the treatment of both acute and chronic bronchitis, as well as chronic coughs (Dong et al., 2024). Table 3 lists some formulas that affect the chemical composition and biological activity of *A.tataricus* (Table 2).

**TABLE 3:** Herbal examination and proof of *A.tataricus*.

Classic	Dynasty	Properties & flavors	Meridian tropism	Efficacy
Shennong Bencao Jing	Qin & Han Dynasties	Bitter, Warm	Not specified	Treats cough with dyspnea, cold-heat accumulation in the chest; eliminates toxins and paralysis; stabilizes the five zang organs
Jingui Yaolüe	Eastern Han Dynasty	Not specified	Not specified	Combined with Shegan (Belamcanda chinensis) and Mahuang (Ephedra sinica) to treat cough and dyspnea caused by wind-cold constraint
Mingyi Bielu	Northern & Southern Dynasties	Not specified	Not specified	Treats cough with purulent blood, relieves palpitations; replenishes deficiency in overexertion syndromes; addresses pediatric convulsions
Xinxiu Bencao	Tang Dynasty	Bitter, Pungent, Warm	Not specified	Treats cough, dyspnea, chest cold-heat stagnation; eliminates toxins and paralysis; stabilizes organs. Also addresses purulent sputum and pediatric convulsions
Yaoxing Lun	Tang Dynasty	Bitter, Neutral	Not specified	Treats consumptive diseases, replenishes deficiency, regulates qi; resolves chest congestion and evil pathogens; addresses fatigue-related heat
Qianjin Fang	Tang Dynasty	Not specified	Not specified	Treats female urinary disorders and hematuria
Rihuazi Bencao	Song Dynasty	Not specified	Not specified	Regulates middle jiao, treats lung atrophy with hematemesis; dissolves phlegm, quenches thirst; moistens skin and nourishes marrow
Zhenglei Bencao	Northern Song Dynasty	Not specified	Not specified	Treats acute throat obstruction (缠喉风) and inability to swallow
Bencao Faming	Ming Dynasty	Not specified	Not specified	Clears and moistens the lungs
Bencao Gangmu	Ming Dynasty	Pungent, Warm, Moistening	Lung Meridian	Essential for lung diseases; moistens without drying. However, excessive use may injure lung yin due to its dispersing nature
Zhangshi Yitong	Qing Dynasty	Not specified	Not specified	Combined with Ejiao, Beimu (Sauromatum diversifolium), Wuweizi (Schisandra chinensis), and Mai Dong (Ophiopogon japonicus) to nourish yin and arrest cough in consumptive lung diseases
Bencao Huiyan	Ming Dynasty	Pungent, Bitter, Warm	Lung Meridian	Clears lung heat, resolves lung qi stagnation; treats consumptive cough
Bencao Shugouyuan	Qing Dynasty	Bitter, Pungent, Warm	Lung Meridian (Blood Level)	Resolves fire-induced stagnation in the lungs; regulates water passages and promotes urination/defecation

## 7 Pharmacology

### 7.1 Anti-inflammatory effect

**FIGURE 13:**
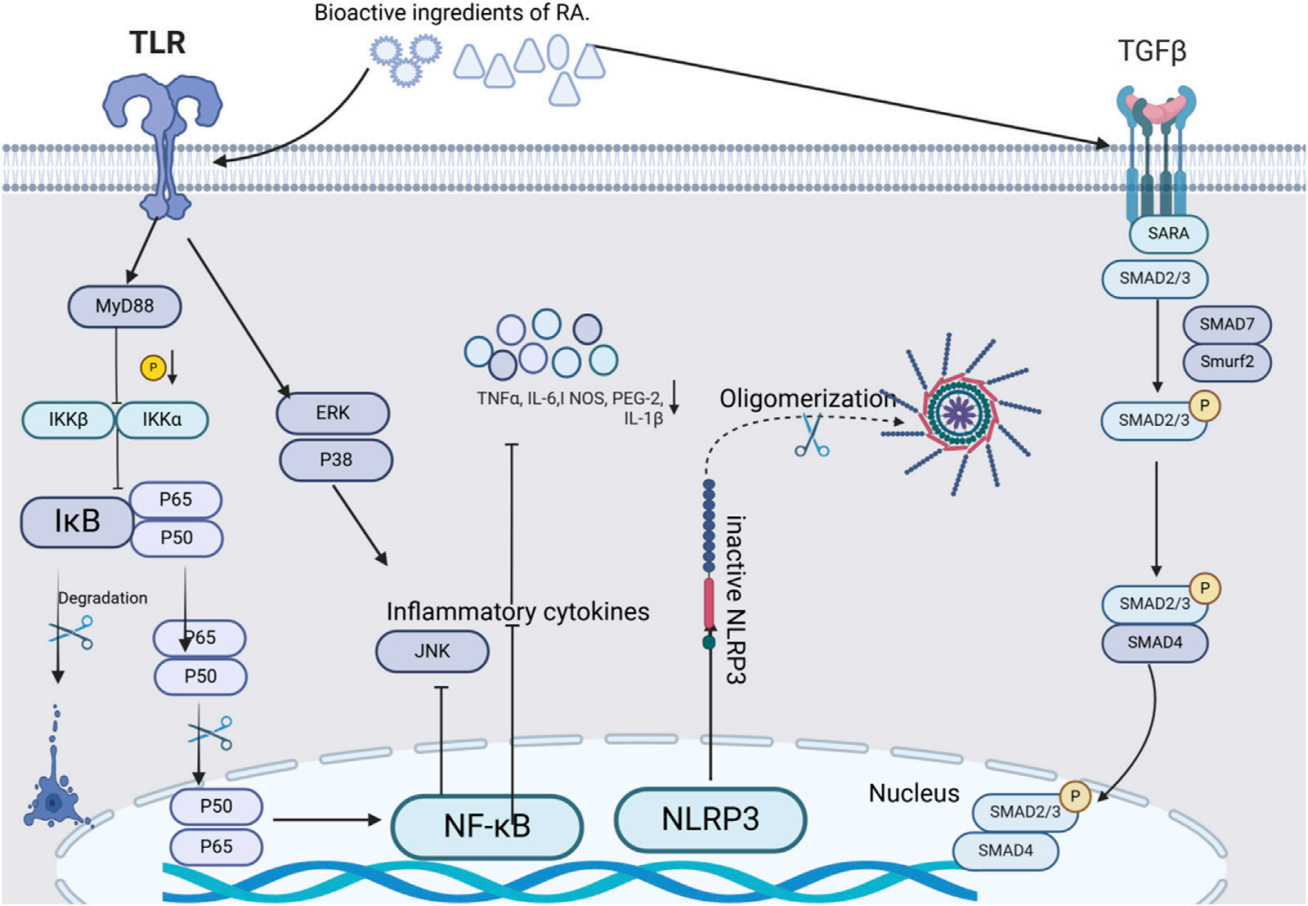
The chief anti-inflammatory, expectorant and antitussive molecular targets and signaling pathways mediated by *A.tataricus* treatment.

Numerous research reports have pointed out that *A.tataricus* has well-established anti-inflammatory activity. Inflammation is a natural self-defense mechanism by which the body initiates protection. It represents a multifaceted physiological mechanism that intricately involves the interplay of the immune system, vascular system, and a diverse array of molecular mediators (Prockop and Oh, 2012). *A.tataricus* mediates inflammatory processes by inhibiting pro-inflammatory mediators, including NO, PGE-2, TNF-α, IL-1β, and IL-6 (Luo et al., 2024). Figure 13 shows a map of the anti-inflammatory mechanisms of *A.tataricus*.

Su et al. (2019b) analyzed the potential inflammatory mechanisms of *A.tataricus* and found that various compounds in the plant inhibited the release of NO from RAW264.7 cells stimulated by LPS. Among these, aster saponin B was significantly more effective than the other compounds. Through inhibition of NF-κB activation significantly reduced NO production in LPS-induced RAW264.7 cells and blocked phosphorylation of JNK, ERK, and p38 in the MAPK pathway. Collectively, these processes result in a substantial anti-inflammatory effect and effectively inhibit the production of inflammatory cytokines (PGE-2, IL-6, and IL-1β). Li et al. (2018) pointed out that astin C inhibits cGASSTING signaling and the innate inflammatory response triggered by cytoplasmic DNA. As an effective active ingredient, 4-HPA in *A.tataricus* inhibits hyperosmotic and hypoxia-induced HIF-1α in NR8383 macrophages and reduces inflammatory cytokine levels (Liu et al., 2014). TGF-β1, a widely studied growth factor has many biological functions, including inflammatory response stimulation. Wang et al. (2022) revealed that the aqueous extract of *A.tataricus* significantly decreased the expression levels of TGF-β1 in the lung tissues of mice infected with *Mycoplasma* pneumoniae.

Shionone exhibits satisfactory anti-inflammatory activity. It diminishes the secretion of TNF-α and IL-1β by inflammatory macrophages. The inhibitory effect was observed to be the same as that of NF-κB inhibitors, both demonstrating a dose-responsive mechanism that curtails NO release (Wang et al., 2015). In their study of TNBS-induced Crohn’s disease (CD) like colitis in mice, Xu et al. found that (Xu et al., 2024) shionone can reduce intestinal inflammatory mediators (TNF-α, IL-6/IL-1β) expression by inhibiting apoptosis of intestinal epithelial cells. Wang X. et al. (2020), Wang et al. (2021) highlighted that in their investigation of interstitial cystitis (IC), *A.tataricus* extract (ATE) demonstrated notable anti-inflammatory effects on both rat bladder tissues and urothelial cells (SV-HUC-1) by inhibiting heat shock protein expression and downregulating NLRP3/GSDMD-N signaling pathway. These effects were particularly pronounced when experimented using ATE. Thus, ATE has a remarkable capability to downregulate NLRP3 and other pyropyretic-associated proteins, suggesting its potential use as an NLRP3 inhibitor for the treatment of IC. Furthermore, shionone functions via the NF-κB/NLRP3/GSDMD-N route, reducing the mRNA and protein quantities of NF-κB, NLRP3, ASC, Pro-caspase-1, Caspase-1, GSDMD, and GSDMD-N, thus disrupting the NLRP3 inflammasome pathway. This facilitates IC occurrence reduction.

### 7.2 Expectorant and antitussive effects

The most common pharmacological effects of *A.tataricus* are its expectorant and antitussive effects. Lu et al. (1999) found that shionone and epifriedelanol have expectorant and antitussive effects. Yang et al. (2008) found that 1-acetoxy-2-ene (E)-4,6-decyne is the main compound in the volatile oil of *A.tataricus* and has expectorant effects. Yu et al. (2015b) confirmed that the expectorant effect of *A.tataricus* is better than that of the anti-cough effect and found that Fr-50, a key component, demonstrates significantly better expectorant effects than shionone. Analysis of Fr-50 revealed that it consisted of 12 chlorogenic acids, 7 saponins, and 13 pentapeptides.

Pharmacopoeia and scholarly documents indicate that *A.tataricus* is primarily used for phlegm removal, cough relief, lung relaxation, and asthma alleviation. Under normal circumstances, coughs are classified as either dry or wet, with different pathogenesis depending on their underlying causes (Asthma Group R. M. B., 2022). Several studies have shown that *A.tataricus* processed with honey has better expectorant and antitussive effects than raw products (Wu et al., 2006). The antitussive effect of *A.tataricus* has two main aspects, as described below.

#### 7.2.1 Anti-inflammatory effect

Sputum and repeated coughing are two typical clinical symptoms of respiratory mucus hypersecretion and chronic non-specific inflammation of surrounding tissues, which can be attributed to chronic inflammatory respiratory diseases (Zhang and Zhou, 2014; Deng et al., 2023). Studies have shown that the TLR4/MyD88/NF-κB pathway is one of the pathogenesis pathways of bronchitis (Wu et al., 2020). Wu et al. (2023) conducted research evaluating the anti-inflammatory properties of *Zi Wan San* (RA and *Tussilago farfara* L), focusing on the TLR4/MyD88/NF-κB signaling pathway. The investigation revealed that administering various dosages of *Zi Wan Sa*n led to significantly reduced TGF-β1, IL-1β, and IL-6 concentrations, alongside a notable decrease in the overall count and types of white blood cells. This reduction was also accompanied by improved lung tissue pathology, lower scores of airway goblet cell proliferation scores, and a decreased in the expression levels of TLR4, MyD88, and NF-κB proteins. Consequently, *A.tataricus* demonstrates the potential to mitigate airway inflammation in chronic bronchitis through its interaction with the TLR4/MyD88/NF-κB signaling pathway. And Ai and Li (2023) found that shionone can reduce reactive oxygen species (ROS) levels, inhibit OVA-induced oxidative stress response and NF-κB signaling pathway in young asthmatic rats, and effectively relieve asthma. Its pharmacological effects are similar to those of dexamethasone.

#### 7.2.2 Vasodilation of bronchial smooth muscles

**FIGURE 14:**
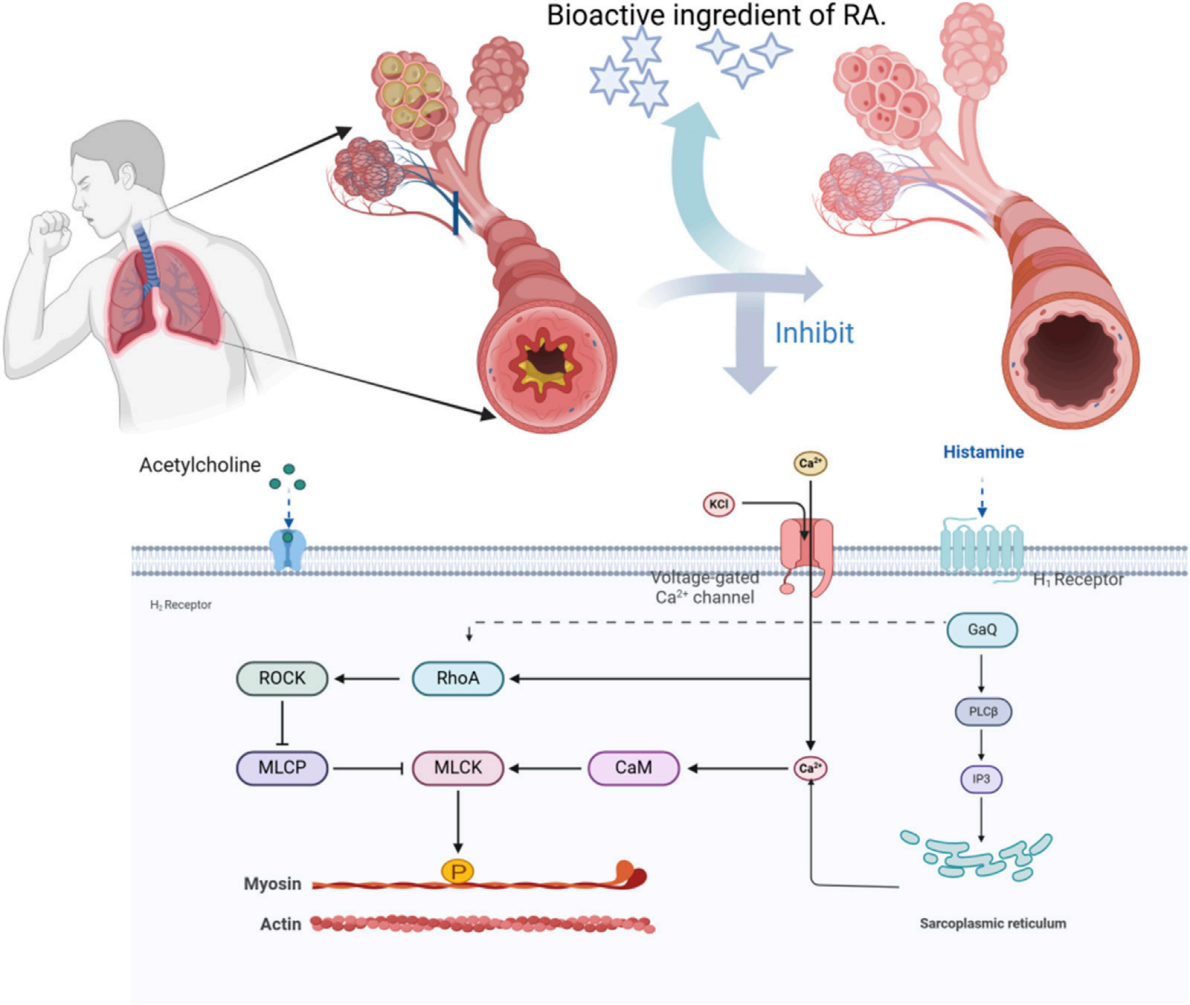
The action mechanism of *A.tataricus* relaxing bronchial smooth muscles.

Cough-variant asthma (CVA) is a special type of asthma with cough as the main symptom and is often chronic and refractory. Irritating dry cough is the main clinical manifestation. In Western medicine, bronchodilators are typically used to treat this condition (Asthma Group R. M. B., 2022). Analysis of 218 prescriptions revealed that *A.tataricus* appeared 205 times during the treatment of CVA (Song C. et al., 2024). Peng et al. (2016a) observed the function of isolated guinea pig trachea smooth muscles and found that high doses of ATEE almost completely inhibited the contraction of isolated guinea pig smooth muscles caused by Ach, histamine, and CaCl_2_. ATEE exhibited a significant non-competitive and coercive effect on these substances, leading to the relaxation of bronchial smooth muscles and exerting an antitussive effect. Chen (2019) verified that ATE eluted using 75% ethanol can not only effectively inhibit the spontaneous contraction of isolated guinea pig tracheal smooth muscle, but also considerably inhibit the contraction of tracheal smooth muscle caused by various agonists, and block the Ca^2+^ channel of smooth muscle cells. It antagonizes the muscarine or histamine receptors on the smooth muscle, inhibits the inflow of Ca^2+^ into the cells, relaxes the lungs, and relieves asthma (Figure 14).

### 7.3 Anti-tumor effect

Bioactive compounds of *A.tataricus* exert inhibitory effects against diverse tumor types, including gastric, lung, prostate, skin, and breast cancers and glioma (Li et al., 2022b). They mainly induce tumor cell apoptosis, inhibit tumor cell proliferation and migration, and maintain the dynamic balance of oxidative stress in the tumor microenvironment.

Gastric cancer, a formidable global health challenge, stands as the predominant cause of cancer-associated deaths worldwide. Zhang Y. et al. (2012) demonstrated that WATP exhibits potent tumor growth inhibitory effects against the human gastric cancer cell line SGC-7901 while maintaining a non-toxic profile. This effect is achieved by inducing apoptosis via Ca^2+^ and △Ψ m-dependent mechanisms. The administration of ATE demonstrates its capability to suppress the Wnt/β-catenin signaling pathway and the expression of VEGF and Ki-67 proteins, thereby inhibiting the proliferation and invasion of lung adenocarcinoma A549 cells. Moreover, it successfully limits the expansion and movement of cancerous tissue while not causing major toxic effects on regular embryonic lung fibroblasts (Yao et al., 2022). In 2021, Chen T. et al. (2022) studied the discovery of a new bioflavonoid compound in *A.tataricus*. This compound primarily inhibited the proliferation and migration of A549 cells, inducing cell death through a non-apoptotic mechanism and thereby inhibiting the spread of lung and pancreatic cells. Liu et al. (2017) demonstrated *in vitro* that different concentrations of honey-processed *A.tataricus* decoction had a specific inhibitory effect on the proliferation of breast cancer MCF-7 cells in the G1 phase. Moreover, a certain concentration of honey-processed *A.tataricus* decoction can also effectively inhibit the migration of colorectal cancer LOVO cells, thereby preventing the recurrence of colorectal cancer (Qian et al., 2015; Yang et al., 2015). Clonogenic ability, an important characteristic of cancer cells, was also targeted, as ATE effectively inhibited the growth of oral squamous cell carcinoma SCC-9 cells by exerting cytotoxicity and anti-clonogenic activity (Wang et al., 2017).

ATP-II in *A.tataricus* effectively inhibited the *in vitro* proliferation of glioma C6 cells. *In vivo*, studies have demonstrated that ATP-II effectively suppresses the growth of glioma C6-implanted tumors and promotes apoptosis in tumor cells by enhancing the Bax/Bcl-2 ratio and stimulating the activities of Caspase-3, 8, and 9. Both strategies led to a robust apoptotic response, characterised by the swift activation of Caspase-3, an effect that was further amplified by the suppression of the survival kinase Akt. This research highlighted that the ability of ATP-II to effectively downregulate Akt effectively represents a distinctive factor contributing to the reduction of tumor growth (Du et al., 2014).

Cyclic polypeptides in *A.tataricus* exhibit desirable anti-tumor effects, and their activity depends on their cyclic nature. Proline dichloride residues prepared via chemical transformation and liver microsomal biotransformation on various homologues sin rats show that 1,2-cis dechlorinated proline residues play an important role in the anti-tumor activity of astin A, B, and C, and have good inhibitory activity against sarcoma 180A (Morita et al., 1996). Studies on the anti-mice ascites tumor cells of astins have shown moderate anti-tumor activity, with the activity of astin A and B being approximately 10 times that of astin C (Morita et al., 1993). The backbones of astin B and astin C were modified using Lawesson’s reagents, and the resulting thioderivatives showed higher anti-tumor activity than the corresponding parent astin (Cozzolino et al., 2005). Moreover, the main compound of the endophytic *bacterium Cyanodermella* astris isolated from *A.tataricus* is the bisanthraquinone skyrin, which has anti-cancer activity (Jahn et al., 2017).

### 7.4 Anti-oxidation and anti-aging effects

Du et al. (2017) found that ATS can enhance the activities of superoxide dismutase (SOD), glutathione peroxidase, and catalase (CAT) in the retinas of streptozotocin-induced diabetic rats and reduced oxidative stress, thus protecting the retina of diabetic rats. Different compounds isolated from different herbs have different effects on combating hemolysis and lipid peroxidation (Yu and Liu, 2009). Ng et al. (2003) showed that quercetin and kaempferol in *A.tataricus* exhibit potent antioxidative capabilities, demonstrating their effectiveness in hindering hemolysis, lipid peroxidation, and the production of superoxide free radicals. Scopoletin and emodin demonstrated efficacies comparable to those of quercetin and kaempferol in the prevention of superoxide radical formation, albeit with lower potency in the inhibition of lipid peroxidation. Aurantiamide acetate exhibited a remarkable ability to reduce superoxide radical formation, whereas 1,7-dihydroxy-6-methylanthraquinone was specifically effective in inhibiting superoxide radical formation alone.

Epifriedelanol, a key component of ATE, demonstrates efficacy in mitigating adriamycin-induced cellular senescence through its ability to suppress SA-β-gal activity and minimize the production of ROS, alongside reducing the activity of p53 protein and the levels of p21 in both human fibroblasts and human umbilical vein endothelial cells (Yang et al., 2011). The family of sirtuin genes, encompassing SIRT1, SIRT2, SIRT3, and SIRT6, participates in cellular activities like repairing DNA damage, aging, and apoptosis and is crucial in reproductive and developmental processes (Wątroba et al., 2017). Ren et al. (2023) has been highlighted by studies involving mature sow oocytes *in vitro* that epifriedelanol when derived from *A.tataricus*, exhibits notable effects. It can augment the antioxidant and anti-apoptotic functions of oocytes by upregulating SIRT family gene expression, thereby extending the *in vitro* culture age of pig oocytes.

### 7.5 Treatment of osteolytic diseases

RANKL and macrophage colony-stimulating factors regulate monocyte differentiation into osteoclasts (Silbermann et al., 2014; Wu et al., 2017; Zou et al., 2008). To investigate the effect of aster shionoside A_2_ on RANKL-induced RAW264.7 cells and Bone Marrow Macrophages (BMM), discovered that administering aster shionoside A2 significantly reduced the phosphorylation levels of ERK1/2, JNK, and p38 proteins, inhibited osteoclast-related gene activation in reaction to RANKL, and impeded the transcription and translation of NFATC1 and c-fos throughout the osteoclast differentiation phase (Su et al., 2022). Subsequently, Lee et al. (2023b) studied the mechanism of ATEE in alleviating osteoporosis, and demonstrated that it significantly inhibited the expression of key osteoclast factor NFATc1, which is induced by RANKL-mediated upregulation of c-Fos and NFATc1 proteins during osteoclast formation. Additionally, ATEE inhibited RANKL expression and RANK-induced osteoclast formation to delay osteoclast differentiation. It also inhibited the expression of RANKL mRNA driven by VitD3 in MLO-Y4 cells, and reduced VitD3-induced osteoclast formation in a co-culture of BMM and MLO-Y4 cells.

### 7.6 Treating urinary system diseases

*A.tataricus* alleviates urological-related diseases and has been clinically proven to have satisfactory effects. There is a record in *Materia Medica Tongxuan*: *A.tataricus* is spicy but not dry, moist but not cold, and tonic but not stagnant. However, its use is typically not immediate. For patients experiencing difficulty in urinating, one or two are recommended for symptom relief (Yu et al., 2023). Clinically, *A.tataricus* has been shown to effectively treat haematuria (Huang, 1990).

ATE safeguards bladder urothelial cells against pyroapoptosis. Through its mechanism of decreasing bladder swelling and bleeding in SD rats, lowering inflammatory markers and histopathological damage, and suppressing the activity of cell proapoptosis-related proteins while highlighting the role of the NLRP3/GSDMD-N pathway, ATE demonstrates a protective function against bladder injury. This leads to an enhancement of cell viability, reduction in the rate of pyroapoptosis in SV-HUC-1 cells, and ultimately, a beneficial effect in mitigating urinary retention, showcasing its therapeutic potential (Wang X. et al., 2020).

Research into the mechanism by which ATE curtails testosterone propionate-induced benign prostatic hyperplasia (BPH) in rats has revealed that *A.tataricus* exhibits therapeutic potential in BPH by enhancing apoptosis and suppressing inflammatory responses, as evidenced by substantial reductions in prostate weight, serum testosterone, and dihydrotestosterone levels, alongside inhibiting prostate epithelial thickening and the upregulation of proliferating cell nuclear antigen in rats. It reduces the expression of Bcl-2, increasing the expression of Bax, and significantly decreases the Bcl-2/Bax ratio. Moreover, it attenuates pro-inflammatory cytokine levels, specifically IL-1β, IL-6, and TNF-α levels, and reduces the expression of COX-2 and iNOS (Rho et al., 2020).

### 7.7 Acute lung injury

Shionone reduced histopathological changes, pulmonary oedema, and MPO activity and increased the percentage of neutrophils and macrophages in BALF. In addition, it inhibited pro-inflammatory cytokines, enhanced anti-inflammatory cytokines, and converted the M1 phenotype into M2 phenotype macrophages. The overexpression of ECM1 *in vivo* and *in vitro* confirmed that the regulatory effect of shionone could be attributed to ECM1 signaling transduction. Shionone weakens the ECM1/STAT5/NF-κB pathway, thereby improving acute lung injury (ALI) caused by sepsis (Song et al., 2021). Another study pointed out that shionone could target pneumohaemolysin *in vivo* and *in vitro*, thereby improving ALI caused by *Streptococcus pneumoniae* (Du et al., 2022). Chen et al. (Chen et al., 2019) analyzed ALI induced by LPS-induced mice using network pharmacology and found that Fr-75 eluted by ATE reduced the number of white blood cells in BALF by 39.2%, macrophages by 51.8%, neutrophils by 63.8%, and lymphocytes by 43.6%. It protects against LPS-induced MPO by reducing the severity of lung lesions, pulmonary oedema, release of inflammatory cells, and expression of ALI. Fr-75 contains many regulatable targets and pathways that may regulate, of which 31 targets are related to multiple components, such as VEGFA and NF-κB pathways. In 2024, research demonstrated that shionone is a viable therapeutic agent for addressing pulmonary inflammation and fibrosis in silicotic mice. Shionone, through its activation of the Nrf2 pathway, safeguards against SO_2_-induced oxidative stress and inflammation in macrophages and prevents fibroblast-to-myofibroblast transition triggered by TGF-β (Wang et al., 2024).

### 7.8 Others

In addition to the above pharmacological effects, Wu et al. (2021) found that *A.tataricus* can relieve constipation by antagonizing the binding of Ach to the AChR and inhibiting Ca^2+^ influx. In South Korea, owing to differences in the growth environment of *A.tataricus*, its leaves have a good recovery effect on memory dysfunction. In their investigation of the pharmacological mechanisms, Lee S. E. et al. (2023) demonstrated that ATE exerts its effects through the modulation of hippocampal cholinergic activity and anti-apoptotic signaling pathways. Specifically, their research revealed that ATE regulates choline acetyltransferase activity and influences the expression of Bcl2-associated anti-apoptotic genes during scopolamine-induced cognitive deficits in murine models. Furthermore, the study identified significant alterations in neuroactive ligand-receptor interaction-related genes, including Npy2r, Htr2c, and Rxfp1. Importantly, ATE administration was shown to mitigate amyloid-β protein aggregation and prevent neuronal apoptosis in transgenic mouse models, resulting in substantial improvements in memory-related cognitive functions.

*In vitro*, ATE exerted a growth-promoting effect on *Bifidobacterium* and an inhibitory effect on *Clostridium perfringens*. Therefore, *A.tataricus* can have a probiotic effect on intestinal flora (Park et al., 1993). Furthermore, terpenoids in *A.tataricus* have certain antiviral activity, especially against HBV. Studies have found that (Zhou et al., 2013) astataricusones B exerts potent antiviral effects through dual mechanisms: primarily by suppressing the secretion of hepatitis B virus surface antigens (HBsAg and HBeAg) and subsequently inhibiting viral DNA replication. Furthermore, pharmacological investigations demonstrated that ATE-derived polyphenolic compounds exhibit remarkable metabolic regulatory properties, as evidenced by their ability to significantly attenuate body weight gain and modulate blood glucose levels in rodent models (Du et al., 2017).

## 8 Acute toxicology

*A.tataricus* has a history of over 2000 years and is generally considered clinically safe. *Chp* recommends a dosage of 5–10 g for *A.tataricus* (Commission N. P, 2020). S. Kosemura et al. (Morita et al., 1993; Yoshioka et al., 1973) found that Astin has a similar structure to that of cyclochloridine, the hepatotoxic metabolite of penicillin. Thus, *A.tataricus* could be toxic and may cause liver damage (Liu et al., 2012). Subsequently, refer to Table 4 for the related toxicity experiment of *A.tataricus*. For the toxicity component screening experiment of *A.tataricus*, the 90% ethanol extract of *A.tataricus* was separated into petroleum ether, ethyl acetate, n-butanol, and water fractions using an extraction method. Using acute toxicity in mice as the indicator, the results showed that the ethyl acetate fraction was the toxic fraction of *A.tataricus*, with an LD_50_ of 0.052 g/kg. After administration of this fraction, the mice exhibited markedly reduced activity, listlessness, unkempt and wet fur, closed eyes, and involuntary tremors until respiratory arrest (Zhang et al., 2007). Through acute toxicity experiments, Shao et al. (2009) showed that ATEE toxicity was greater than that of ATE, with toxicity levels increasing as ethanol concentration increased. Among the tested ethanol concentrations, 90% ethanol extract was found to be the most toxic. Acute toxicity tests were conducted on the water extract, 75% ethanol extract, and 80% ethanol extract of *A.tataricus*. Most mice exhibited abnormal behaviors post-administration, such as delayed movement, listlessness, and refusal to eat. The LD_50_ values were 31.61 g/kg, 15.74 g/(kg·bw), and 19.19 g/kg, respectively. For the 75% ethanol extract-treated mice, autopsy revealed darkened liver color, blunt margins, and obvious swelling, congestion, spots, and bleeding points (Liu et al., 2013; Peng, 2016). The toxicity in ATEE mainly came from the cancer fraction Fr-2 compound, which increased the levels of aminotransferase, aspartate aminotransferase, and alkaline phosphatase. A histopathological examination of the liver confirmed that the compound is hepatotoxic. Subsequent screening and analysis of the hepatotoxic compounds revealed that Fr-2 is primarily a peptide compounds (Liu et al., 2012). Fr-2 induces toxicity in human L-02 liver cells via oxidative stress. Intracellular ROS levels peak at 40 μg/mL. It triggers liver cell apoptosis through a mitochondrial-dependent pathway in a dose-dependent manner. At 100 μg/mL, the mitochondrial membrane potential drops from 5.29% to 39.83%, and apoptosis-related proteins are activated (Wang et al., 2014b). This is consistent with a previous discovery that Astin B can induce apoptosis and autophagy in human liver L-02 cells and is mediated by mitochondrial/caspase-dependent apoptosis *in vivo* and *in vitro*, showing hepatotoxicity results (Wang et al., 2014a). Studies on the combined toxicity of *A.tataricus* and *T.farfara* show that combining different solvent extracts of *T.farfara* with the 90% ethanol extract of *A.tataricus* in a 1:1 ratio by raw material weight can significantly reduce liver damage caused by *A.tataricus* (Zhang et al., 2007 *)*. In addition to cyclic peptides, 11 anti-HBV shionane-type triterpenoids in *A.tataricus* were studied to identify that astaricusones B, epifriedelan, and astershionones C were cytotoxic to HepG2.2.15 cells (Zhou et al., 2013; Zhou et al., 2014). The saponins in *A.tataricus* have a hemolytic effect, so its crude extract is not suitable for intravenous use (Wang, 1983).

**TABLE 4:** Acute toxicity experiment.

Experimental materials	Toxicological effect	Dose level	Experimental model	LD50	Risk factors	References
75% ethanol extract	Acute toxicity reaction	9.88–25.08 g/(kg·bw)	Kunming mice	15.74 g/(kg·bw)	High - dose use may cause acute toxicity, especially to the liver	Peng et al. (2016a)
Different polarity substances	Subchronic toxicity reaction	0.34 g/(kg·bw)	SD rats	-	Long - term use may lead to liver damage, particularly with petroleum ether and ethyl acetate extracts	Peng (2016)
Fr-2 fraction	Hepatotoxicity Experiment	20–100 μg/mL	Human liver cells L-02	-	Oxidative stress induces toxicity in human L - 02 liver cells, triggering apoptosis via a mitochondrial - dependent pathway in a dose - dependent manner	Wang et al. (2014b)
Water extract	Acute toxicity reaction	23.7–36 g/kg	Kunming mice	31.61 g/kg	High - dose use may result in acute toxicity, while low - dose use can normalize conditions in 3 days	Liu et al. (2013)
80% alcohol extract	Acute toxicity reaction	12.5–31.6 g/kg	Kunming mice	19.19 g/kg	High - dose use may result in acute toxicity, while low - dose use can normalize conditions in 3 days	Liu et al. (2013)
Fr-2 fraction	Acute toxicity reaction	0.023–0.1 g/kg	Kunming mice	0.052 g/kg	Low doses cause mild liver injury in mice, while high doses can lead to severe acute liver injury and death	Wang et al. (2010)

Although extensively used in traditional medicine and generally considered safe, *A.tataricus* has potential hepatotoxicity and hemolytic properties that should not be overlooked. Its toxic effects are more pronounced at high doses or with long-term use. To ensure its clinical safety, more comprehensive and reliable data are needed to clarify its potential toxicity and explore more effective detoxification methods. Additionally, combining *A.tataricus* with other Chinese herbs like Tussilago farfara could be an effective way to reduce toxicity.

## 9 Comprehensive utilization

### 9.1 Traditional food applications

In countries such as China, North Korea, and Japan, *A.tataricus* is often consumed as a vegetable. According to documents issued by the *National Health Commission of China* and the *State Administration of Market Supervision of China*, *A.tataricus* is approved for use as both medicine and food within a limited scope of use and dose (Wang et al., 2018). Generally, young leaves harvested in the spring are generally consumed as vegetables. Fresh vegetables can be washed, blanched with boiling water, and soaked in clear water overnight. They can be dried and left for later consumption as dried vegetables, soaked and eaten directly as soups or dipped in sauce, and soaked in scented tea and made into *Zi Wan* tea (Chen and Huang, 2010). In South Korea, *A.tataricus* is among the most popular wild vegetables (Ahn et al., 2018) used to make traditional Korean dishes (Chung et al., 1993).

### 9.2 Essential oils

The concentrated essential oil of *A.tataricus* maintains the same taste as that of fresh plants, with a woody or herbal aroma (Choi, 2012). Chung et al. (1993) determined that monoterpenes and sesquiterpenes are the primary volatile components in *A.tataricus* extracts. Among these, myrene, limonene, and germanene D were identified as the predominant volatile compounds within *A.tataricus* essential oils.

### 9.3 Ornamental plants

*A.tataricus* can be used as an ornamental flower in the autumn. When its lavender flowers are in full bloom, they contrast the high and dark blue sky in autumn and have an extremely high ornamental value. In the gardens of European and American countries, *A.tataricus* is an important cultivated variety, in China, it is recommended for use in flowerbeds due to its rapid growth, ease of management, light preference, and drought tolerance (Yang, 2003). In South Korea and Japan, *A.tataricus* is a common autumn wildflower (Lee et al., 2021). For this reason, Japan has also developed the unique color of Aster-shion-iron (dyed by RA and *Camellia japonica* gray juice as a mordant). Considered a symbol of peace (Chen, 1985).

### 9.4 Others

*Clonorchiasis sinensis* is an important food-borne zoonotic parasite, highly associated with liver fibrosis and cholangiocarcinoma (Bao et al., 2024). Bao et al. (2024) observed the insecticidal effects of ATE on *Clonorchiasis sinensis in vitro*.

Additionally, oxidative rancidity is the main reason for the deterioration of the nutritional quality of grease and oily foods (Hu et al., 2022). Chen et al. (2012) found that ATE has a good antioxidant effect on peanut oil, and the increase in the concentration of the extract is positively correlated with the anti-oxidation effect of peanut oil. Therefore, the *A.tataricus* extract can be used instead of artificial antioxidants, which can not only extend the life of oil products, but also be harmless to human health.

## 10 Conclusion and prospects

Medicinal plants are esteemed for their pivotal role in human health, serving as a fundamental source of bioactive compounds that are harnessed to prevent, manage, and cure a wide array of common ailments (Dsouza et al., 2024). *A.tataricus* has a variety of chemical components, so its medicinal value is very high. As a medicinal and edible plant, *A.tataricus* has been used for over 1700 years since its medicinal effects were discovered. Ethnocpharmacology believes that it mainly has the effects of warming the lungs, lowering the qi, eliminating phlegm, and relieving cough. Modern clinical research has demonstrated its therapeutic effect against inflammatory diseases, particularly as an expectorant and antitussive. It is the preferred choice for many pharmaceutical companies to produce drugs to moisten the lungs, eliminate phlegm, relieve coughs, and treat asthma (Wei et al., 2023).

Although the root of *A.tataricus* is found in many proprietary Chinese medicines for relieving cough and asthma, with the progress of science and technology, modern studies have revealed that it contains more than 200 kinds of natural metabolites, among which triterpenoids (such as the iconic component shionone), flavonoids and cyclic peptides play anti-inflammatory, antioxidant and anti-tumor effects through multi-target synergies. Notably, *ChP* has designated shionone as a quality marker due to its role as a core anti-inflammatory component in *A.tataricus*, and the underlying mechanism has been extensively elucidated. Cyclopeptides exhibit “double-edged sword” properties, demonstrating significant anticancer effects; however, they share structural similarities with cyclochloroside, a hepatotoxic metabolite of penicillin, which can cause liver damage. Toxicological studies have also explored the mechanisms of liver damage associated with astin. To ensure clinical safety, more comprehensive and reliable data are required to clarify potential toxicity and develop more effective detoxification methods. Further research is essential to understand the efficacy of A.tataricus and its bioactive compounds fully. For instance, the antiviral properties of *A.tataricus* warrant additional validation. This will provide a novel model for integrating traditional medical theory with modern pharmacology.

As a natural herb, *A.tataricus* possesses edible aerial parts and valuable underground medicinal components, both of which exhibit excellent health benefits and thus represent a promising resource for the development of functional foods and innovative medicines. In this paper, we have reviewed the planting methods of *A.tataricus*; however, current research on its cultivation remains limited, and studies exploring the relationship between planting techniques and pharmacological activity are scarce. Factors such as the origin (Xiu et al., 2006), harvest period (Zhang et al., 2021), processing methods (Wang J. et al., 2023), and storage conditions of *A.tataricus* may lead to potential ‘deterioration’. To investigate the effects of planting time, density, and environmental factors on *A.tataricus*, further research is needed to ensure high-quality and stable sustainable development. Additionally, integrating network pharmacology with multi-omics approaches can facilitate the exploration of innovative drugs derived from *A.tataricus*, thereby promoting the modernization of *A.tataricus* research (Marmouzi et al., 2021; Zhang et al., 2024). In summary, this paper comprehensively reviews and analyzes botany, cultivation techniques, phytochemistry, ethnopharmacology, pharmacology, and comprehensive utilization of *A.tataricus*, providing valuable insights for its further development.

## References

[B1] Administration, C. F. A. D. (1999). Chinese materia madica.

[B2] Ahn S. H. Moon J. S. Lee Y. M. Yang J. H. Kim J. Kim H. J. (2018). (Year) published. Selection of suitable wild herbs and vegetables for cultivation of sub-highland. Proceedings of the plant resources society of Korea conference. Plant Resour. Soc. Korea, 53.

[B3] Ai K. Li Z. (2023). -The effects of shionone on nf-κb pathway and oxidative stress. Ovalbumin-Induced Asthmatic Immature Rats. Curr. Immunol. 43, 226–232.

[B4] Akihisa T. Kimura Y. Tai T. Arai K. J. C. Bulletin P. (1999). Astertarone B, a hydroxy-triterpenoid ketone from the roots of aster tataricus L. A Hydroxy-Triterpenoid Ket. Roots Of Aster Tataricus L 47, 1161–1163. 10.1248/cpb.47.1161

[B5] Asthma Group, R. M. B. (2022). Chinese medical association. Guidelines for the diagnosis and treatment of cough (2021). Chin. J. Of Tuberc. And Respir. 45, 13–46.

[B6] Bao P. Wang X. Zhang X. Yu Y. Ma Y. Zhang H. (2024). Clonorchis sinensis aggravated liver fibrosis by activating parp-1 signaling to induce parthanatos via dna damage. Vet. Parasitol. 330, 110217. 10.1016/j.vetpar.2024.110217 38861911

[B7] Cai Y. Shi X. Liu H. Wu L. Gu Y. Wang R. (2023). Eff. Components And Pharmacol. Eff. Of Asteris Radix J. Of Traditional Chin. Veterinary Med. 42, 39–42. 10.13823/J.Cnki.Jtcvm.2023.039

[B8] Chen R. Liu Y. F. Huo L. N. (2012). Antioxidant activity of extracts from aster tataricus. L.F. For oil technology and development of chemical industry, 41, 4–6.

[B9] Chen T. Yang P. Chen H. J. Huang B. (2022a). A new biflavonoids from aster tataricus induced non-apoptotic cell death in A549 cells. Nat. Prod. Res. 36, 1409–1415. 10.1080/14786419.2021.1882456 33615932

[B10] Chen W. Yang Y. Fu K. Zhang D. Wang Z. (2022b). Progress in icp-ms analysis of minerals and heavy metals in traditional medicine. Front. Pharmacol. 13, 891273. 10.3389/Fphar.2022.891273 35837276 PMC9274010

[B11] Chen W. Z. Guo W. N. Wang R. (2020). “Study on the dynamic accumulation of chief chemical constituents of aster tataricus L,” in F. ’Underground organs, 42. Journal Of Yichun University, 27–30.

[B12] Chen Y. Dong J. Liu J. Xu W. Wei Z. Li Y. (2019). Network pharmacology-based investigation of protective mechanism of aster tataricus on lipopolysaccharide-induced acute lung injury. Int. J. Mol. Sci. 20, 543. 10.3390/Ijms20030543 30696024 PMC6387216

[B13] Chen Y. J. (2019). “Study on the pharmacodynamic material basis and mechanism of aster tataricus L. F. On moistening lung and relieving asthma,” in Doctor's degree. Beijing University Of Chinese Medicine.

[B14] Chen Y. L. (1985). Wild flowers aster tataricus L. F. Are beautiful.

[B15] Chen Y. M. Huang H. B. (2010). Health protection and treatment of aster tataricuse L. F. Chin. Med. And Cult. 5, 47.

[B16] Cheng D. Shao Y. (1994). Terpenoid glycosides from the roots of aster tataricus. Phytochemistry 35, 173–176. 7764375

[B17] Choi H.-S. J. A. C. L. (2012). Comparison of the essential oil composition between*Aster tataricus*and*A. Koraiensis* . Koraiensis 2, 138–151. 10.1080/22297928.2000.10648262

[B18] Chung T. Y. Eiserich J. P. Shibamoto T. (1993). Volatile Compd. Isol. Edible Korean Chamchwi (Aster Scaber Thunb) 41, 1693–1697.

[B19] Commission, J. P (2023). Pharmacopoeia of Japan publishing Co., ltd. Tokyo: Japanese Pharmacopoeia.

[B20] Commission, N. P. (2020). Pharmacopoeia of the People's Republic of China, China China National Pharmaceutical Science And Technology Press.

[B21] Cozzolino R. Palladino P. Rossi F. Calì G. Benedetti E. Laccetti P. (2005). Antineoplastic cyclic astin analogues kill tumour cells via caspase-mediated induction of apoptosis. Carcinogenesis 26, 733–739. 10.1093/Carcin/Bgi017 15661812

[B22] Deng Z. Liu Y. Zhou S. Liu F. (2023). Master of traditional Chinese medicine Liu zuyi's experience in. Treat. And Prev. Chronic Bronchitis Asia-Pacific Traditional Med. 19, 92–94.

[B23] Ding Z. (2021). “Study on geographical identification and the element distribution of Poria cocos based on multi-element combined chemometrics,” in Master's degree master's degree. Anhui University Of Chinese Medicine.

[B24] Dong Y. Liu Y. Tang J. Du J. Zhuang X. Tan S. (2024). Zhisou powder displays therapeutic effect on chronic bronchitis through inhibiting pi3k/akt/hif-1α/vegfa signaling pathway and reprograming metabolic pathway of arachidonic acid. J. Ethnopharmacol. 319, 117110. 10.1016/J.Jep.2023.117110 37673198

[B25] Dsouza A. Dixon M. Shukla M. Graham T. J. J. O. E. B. (2024). Harnessing controlled environment systems for enhanced production of medicinal plants. Erae248. 10.1093/jxb/erae248 PMC11659182 38814918

[B26] Du H. Zhang M. Yao K. Hu Z. (2017). Protective effect of aster tataricus extract on retinal damage on the virtue of its antioxidant and anti-inflammatory effect in diabetic rat. Biomed. Pharmacother. 89, 617–622. 10.1016/J.Biopha.2017.01.179 28262614

[B27] Du L. Mei H. F. Yin X. Xing Y. Q. (2014). Delayed growth of glioma by A polysaccharide from aster tataricus involve upregulation of bax/bcl-2 ratio, activation of caspase-3/8/9, and downregulation of the Akt. Tumor Biol. 35, 1819–1825. 10.1007/S13277-013-1243-8 24081677

[B28] Du R. Wang T. Lv H. Zou Y. Hou X. Hou N. (2022). Shionone-targeted pneumolysin to ameliorate acute lung injury induced by Streptococcus pneumoniae *in vivo* and In Vitro. Molecules 27, 6258. 10.3390/Molecules27196258 36234795 PMC9573397

[B29] Fan L. Wang X. Zhu X. J. Liu J. Yin H. Luo J. P. (2018). Effects of different processing methods on content of extract and main components of A.tataricus. Mod. Chin. Med. 20, 1509–1514. 10.13313/J.Issn.1673-4890.20180630002

[B30] Fan X. Qin Z. Wen J. Wang Z. Xiao W. (2024). An updated and comprehensive review of the morphology, ethnomedicinal uses, phytochemistry, and pharmacological activity of aster tataricus L. F. Heliyon 10, E35267. 10.1016/J.Heliyon.2024.E35267 39166058 PMC11334675

[B31] Gao J. H. Wang H. W. Song G. Q. (1994). Structure and stereochemistry analysis of two compounds from aster tataricus by nmr. Chin. J. Of Magnetic Reson., 391–398.

[B32] Gao W. Y. Zhang R. Jia W. J P. L. Lu B. Zheng Z. Y. (2003). Determination of shionone content in root of aster tataricus by hplc Chinese traditional and herbal drugs, 92–93.

[B33] Guo W. N. Cheng L. Fang C. W. (2016). Studies on Structure,Accumulation of chief chemical constituents and content of aster tataricus L. F.Maternal root. Lishizhen Med. And Materia Medica Res. 27, 2614–2616.

[B34] Han Y. Gao F. Qin P. Bai R. Tian J. Liu X. (2023). Advances in studies on asteris radix et rhizoma and prediction of its quality markers. Mod. Chin. Med. 25, 655–664. 10.13313/J.Issn.1673-4890.20211115004

[B35] Hu G. (2017). “Study on inorganic elements and quality analysis of polygonum cuspidatum in shitai of Anhui Province,” in Master’s degree. Anhui University Of Traditional Chinese Medicine.

[B36] Hu W. N. Xue Y. T. Wang X. L. Hao X. Q. Wang Z. Q. Song M. P. (2022). Research Progress In Edible oil storage modern chemical research, 16–18.

[B37] Hu Y. Q. (2014). Eco-friendly cultivation technology for aster tataricus. Hebei Agric., 20–21.

[B38] Huang L. Q. Guo L. P. Zhan Z. L. (2020). Compendium of standards for genuine medicinal materials. Beijing: Beijing Science And Technology Press.

[B39] Huang M. (1990). Reuse aster to treat blood in. Urine Journal Of Shanxi University Of Traditional Chinese Medicine, 26.

[B40] Huang S. S. Gao Y. Li W. M. Guan H. H. (2008). Determination of total triterpenes from radix aster is by spectrophoto metry lishizhen medicine and materia Medica research, 1406–1407.

[B41] Jahn L. Schafhauser T. Wibberg D. Rueckert C. Winkler A. Kulik A. (2017). Linking secondary metabolites to biosynthesis genes in the fungal endophyte Cyanodermella asteris: the anti-cancer bisanthraquinone skyrin. Anti-Cancer Bisanthraquinone Skyrin 257, 233–239. 10.1016/j.jbiotec.2017.06.410 28647529

[B42] Jaiswal V. Lee H. J. (2023). Pharmacological properties of shionone: potential anti-inflammatory phytochemical against different diseases. Molecules 29, 189. 10.3390/Molecules29010189 38202771 PMC10780092

[B43] Jia K. Zhang X. Meng Y. Liu S. Liu X. Yang T. (2023). Metabolomics and transcriptomics provide insights into the flavonoid biosynthesis pathway in the roots of developing aster tataricus. J. Plant Res. 136, 139–156. 10.1007/S10265-022-01426-4 36520245 PMC9753034

[B44] Jia S. X. (2015). Transforming the significance of medicinal substances, zhongguo zhong Yi yao chu ban she.

[B45] Jin J. Moore M. K. Wilson W. K. Matsuda S. P. T. (2018). Astertarone A synthase from Chinese cabbage does not produce the C4-epimer: mechanistic insights. Org. Lett. 20, 1802–1805. 10.1021/Acs.Orglett.8b00302 29557662

[B46] Jin J. Zhang C. F. Zhang M. (2008). Study on chemical constituents from aster tataricus L.F. Mod. Chin. Med., 20–22. 10.13313/J.Issn.1673-4890.2008.06.012

[B47] Kang K. B. Lee D. Y. Sung S. H. (2021). LC–MS/MS-Based comparative investigation on chemical constituents of six aster species occurring in Korea. Korea. Nat. Product. Sci. 27, 257–263. 10.20307/nps.2021.27.4.257

[B48] K., D. (2023). Aster, A neglected family of compositae flowers China flowers and horticulture, 30–33.

[B49] Lanzotti V. J. P. R. (2005). Bioactive saponins from allium and aster plants. Bioact. Saponins Allium And Aster Plants 4, 95–110. 10.1007/s11101-005-1254-1

[B50] Lee J. S. Lee C. K. Choi Y. J. Shin H. D. Reports J. N. D. (2021). Pucciniastrum verruculosum causing rust disease on aster tataricus in Korea. 44, Na-Na.

[B51] Lee S. E. Park S. Jang G. Y. Lee J. Moon M. Ji Y. J. (2023a). Extract of aster koraiensis nakai leaf ameliorates memory dysfunction via anti-inflammatory action. Int. J. Mol. Sci. 24, 5765. 10.3390/ijms24065765 36982837 PMC10052554

[B52] Lee S. J. Yang H. Kim S. C. Gu D. R. Ryuk J. A. Jang S. A. (2023b). Ethanol extract of radix asteris suppresses osteoclast differentiation and alleviates osteoporosis. Int. J. Mol. Sci. 24, 16526. 10.3390/Ijms242216526 38003715 PMC10671772

[B53] Li H. Li W. Ding X. Zhang D. Xue Z. An Q. (2024a). Herbal textual research on asteris radix et rhizoma. Famous Class. Formulas Chin. J. Of Exp. Traditional Med. Formulae 30, 20–30. 10.13422/J.Cnki.Syfjx.20230356

[B54] Li H. Y. Guo S. Yan H. Yang T. Yu D. X. Zhan Z. L. (2022a). Content and distribution of inorganic elements in laminaria japonica based on icp-ms and micro-xrf. Zhongguo Zhong Yao Za Zhi 47, 444–452. 10.19540/J.Cnki.Cjcmm.20210803.203 35178988

[B55] Li J. Chen C. Ma M. Li P. Yang S. Guo R. (2024b). Five new secondary metabolites from aster tataricus. Fitoterapia 174, 105828. 10.1016/J.Fitote.2024.105828 38296166

[B56] Li K. J. Liu Y. Y. Wang D. Yan P. Z. Lu D. C. Zhao D. S. (2022b). Radix asteris: traditional usage, phytochemistry and pharmacology of an important traditional Chinese medicine. Molecules 27, 5388. 10.3390/Molecules27175388 36080154 PMC9458035

[B57] Li L. Chang X. Cui H. Sun H. Cao T. (2023a). Comparative analysis of inorganic elements in difference officinal parts of aster tataricus by icp-ms with statistical analysis. J. Of Chin. Med. Mater. 46, 1447–1453. 10.13863/J.Issn1001-4454.2023.06.021

[B58] Li S. Hong Z. Wang Z. Li F. Mei J. Huang L. (2018). The cyclopeptide astin C specifically inhibits the innate immune cdn sensor sting. Cell Rep. 25, 3405–3421. 10.1016/J.Celrep.2018.11.097 30566866

[B59] Li S. Z. (2012). The Compendium of materia Medica, People’s medical publishing house.

[B60] Li X. Gong Y. Feng L. Wang X. Wang J. Zhang A. (2023b). Neuropyrones A-E, five undescribed Α-pyrone derivatives with tyrosinase inhibitory activity from the endophytic fungus neurospora dictyophora wz-497. Phytochemistry 207, 113579. 10.1016/J.Phytochem.2022.113579 36586529

[B61] Li Y. Luo K. P. Chen Y. Y. Lin Y. Chen Q. B. (2010). A brief analysis of the current research situation of aster tataricus in China. Guangxi J. Of Light Industry 26, 7–8.

[B62] Liu F. Li L. F. Liu G. J. Jiang H. Zhang Q. Z. (2013). Study on the acute toxicity of water and alcohol extract of radix asteris. Res. And Pract. Chin. Med. 27, 38–39. 10.13728/J.1673-6427.2013.06.018

[B63] Liu W. Feng Y. Yu S. Fan Z. Li X. Li J. (2021). The flavonoid biosynthesis network in plants. Int. J. Mol. Sci. 22, 12824. 10.3390/Ijms222312824 34884627 PMC8657439

[B64] Liu X. D. Cao P. P. Zhang C. F. Xu X. H. Zhang M. Pharmaceutical J. J. O. (2012). Screening and analyzing potential hepatotoxic compounds in. Ethanol Extr. Of Asteris Radix By Hplc/Dad/Esi-Msn Tech. 67, 51–62. 10.1016/j.jpba.2012.04.034 22609370

[B65] Liu X. L. Liang Y. X. Hu X. H. (2017). Impacts of the decoction of honey processed radix asteris on human breast cancer mcf-7 cells in. Intro Experimen World J. Of Integr. Traditional And West. Med. 12, 496–499+512. 10.13935/J.Cnki.Sjzx.170412

[B66] Liu Z. Xi R. Zhang Z. Li W. Liu Y. Jin F. (2014). 4-hydroxyphenylacetic acid attenuated inflammation and edema via suppressing HIF-1α in seawater aspiration-induced lung injury in rats. Rats. Int J Mol Sci 15, 12861–12884. 10.3390/Ijms150712861 25050781 PMC4139878

[B67] Lu Y. H. Dai Y. Wang Z. T. Xu L. S. (1999). Aster tataricuse for expectorant and antitussive use, and its effective parts and active ingredients. Chin. Herb. Med., 360–362.

[B68] Lu Y. H. Wang Z. T. Ye W. C. Xu L. S. Shu Y. Z. (1998). Studies on the constituents of aster tataricus L.F. Journal of China pharmaceutical university, 19–21.

[B69] Luo M. Zhao F. Cheng H. Su M. Wang Y. (2024). Macrophage polarization: an important role in inflammatory diseases. Front. Immunol. 15, 1352946. 10.3389/Fimmu.2024.1352946 38660308 PMC11039887

[B70] Marmouzi I. Bouyahya A. Ezzat S. M. El Jemli M. Kharbach M. J. J. O. E. (2021). The food plant Silybum marianum (L.) Gaertn.: phytochemistry, Ethnopharmacology and clinical evidence. Ethnopharmacol. And Clin. Evid. 265, 113303. 10.1016/j.jep.2020.113303 32877720

[B71] Morita H. Nagashima S. Takeya K. Itokawa H. (1993). Astins A and B, antitumor cyclic pentapeptides from aster tataricus. Chem. Pharm. Bull. (Tokyo) 41, 992–993. 10.1248/Cpb.41.992 8339347

[B72] Morita H. Nagashima S. Uchiumi Y. Kuroki O. Takeya K. Itokawa H. (1996). Cyclic peptides from higher plants. Xxviii. Antitumor activity and hepatic microsomal biotransformation of cyclic pentapeptides, astins, from aster tataricus. Chem. Pharm. Bull. (Tokyo) 44, 1026–1032. 10.1248/Cpb.44.1026 8689717

[B73] Morris S. M. Jr (2016). Arginine metabolism revisited. J. Nutr. 146, 2579S–2586S. 10.3945/Jn.115.226621 27934648

[B74] Nagao T. Hachiyama S. Okabe H. Yamauchi T. (1989). Stud. Const. Of Aster Tataricus Lf Ii. Struct. Of Aster Saponins Isol. Root 37, 1977–1983.

[B75] Ng T. B. Liu F. Lu Y. Cheng C. H. Wang Z. (2003). Antioxidant activity of compounds from the medicinal herb aster tataricus. Comp. Biochem. Physiol. C Toxicol. Pharmacol. 136, 109–115. 10.1016/S1532-0456(03)00170-4 14559292

[B76] Park J. H. Han N. S. Yoo J. Y. Kwon D. J. (1993). Effect Of Aster Scaber Extract On The Growth Of Bifidobacteria And Clostridium Perfringens 3, 285–291.

[B77] Peng W. J. (2016). Studies on isolated Guinea-pig tracheal smooth muscle and toxic effects of aster tataricus L. F. Master, Chin. Acad. Of Agric. Sci. Diss.

[B78] Peng W. J. Xin R. H. Liu Y. Luo Y. J. Wang G. B. Luo C. Y. (2016a). Effects of alcohol extract of aster tataricus L.F. Contract. Of Guin. Pig Tracheal Smooth Muscle China Animal Husb. And Veterinary Med. 43, 1572–1578. 10.16431/J.Cnki.1671-7236.2016.06.026

[B79] Peng W. J. Xin R. H. Luo Y. J. Liang G. Ren L. H. Liu Y. (2016b). Evaluation of the acute and subchronic toxicity of *Aster tataricus* L. F. Afr. J. Tradit. Complement. Altern. Med. 13, 38–53. 10.21010/Ajtcam.V13i6.8 PMC5412200 28480359

[B80] Prockop D. J. Oh J. Y. (2012). Mesenchymal stem/stromal cells (mscs): role as guardians of inflammation. Mol. Ther. 20, 14–20. 10.1038/Mt.2011.211 22008910 PMC3255583

[B81] Qian S. S. Peng L. Yang Y. Y. Qin T. T. Liu L. J. Bai S. M. (2015). Effects of honey-fried radix asteris decoction (hfrad) on cell apoptosis and migration in. Colorectal Cancer Lovo Cells 33, 2146–2150. 10.13193/J.Issn.1673-7717.2015.09.027

[B82] Ren X. Yun X. Yang T. Xu T. Shi D. Li X. (2023). Epifriedelanol delays the aging of porcine oocytes matured invitro. Vitro 233, 107256. 10.1016/j.toxicon.2023.107256 37586610

[B83] Rhee J. K. Woo K. J. Baek B. K. Ahn B. J. (1981). Screening of the wormicidal Chinese raw drugs on Clonorchis sinensis. Screen. Of Wormicidal Chin. Raw Drugs Clonorchis Sinensis 9, 277–284. 10.1142/s0192415x81000366 6764091

[B84] Rho J. Seo C. S. Park H. S. Jeong H. Y. Moon O. S. Seo Y. W. (2020). Asteris radix et rhizoma suppresses testosterone-induced benign prostatic hyperplasia in rats by regulating apoptosis and inflammation. J. Ethnopharmacol. 255, 112779. 10.1016/J.Jep.2020.112779 32209388

[B85] Ri H. Z. Shang Z. J. (1983). Rihuazi bencao, southern Anhui medical College.

[B86] Rossi F. Zanotti G. Saviano M. Iacovino R. Palladino P. Saviano G. (2004). New antitumour cyclic astin analogues: synthesis, conformation and bioactivity. J. Pept. Sci. 10, 92–102. 10.1002/Psc.506 14994987

[B87] Sawai S. Uchiyama H. Mizuno S. Aoki T. Akashi T. Ayabe S. (2011). Molecular characterization of an oxidosqualene cyclase that yields shionone, A unique tetracyclic triterpene ketone of aster tataricus. Febs Lett. 585, 1031–1036. 10.1016/J.Febslet.2011.02.037 21377465

[B88] Schafhauser T. Jahn L. Kirchner N. Kulik A. Flor L. Lang A. (2019). Antitumor astins originate from the fungal endophyte *Cyanodermella asteris* living within the medicinal plant *Aster tataricus* . Antitumor Astins Orig. Fungal Endophyte Cyanodermella Asteris Living Within Med. Plant Aster Tataricus 116, 26909–26917. 10.1073/pnas.1910527116 PMC6936678 31811021

[B89] Shao J. Jin J. Zhang M. Huang F. Dou C. G. Wei Y. Y. (2009). Toxic Part Of radix asteris and antagonizing Part Of flos farfarae in pair-drug of ff and Ra. Lishizhen Med. And Materia Medica Res. 20, 1308–1310.

[B90] Silbermann R. Bolzoni M. Storti P. Guasco D. Bonomini S. Zhou D. (2014). Bone Marrow monocyte-/macrophage-derived activin A mediates the osteoclastogenic effect of il-3 in multiple myeloma. Leukemia 28, 951–954. 10.1038/Leu.2013.385 24369304 PMC3981881

[B91] Song C. Yu S. Zhou Y. (2024a). The experience of traditional Chinese medicine in treating cough variant asthma was discussed based on data mining technology. Asia-Pacific Tradit. Med. 20, 196–201.

[B92] Song L. Hou J. P. Li H. B. Zhang Y. G. Y. X. (2009). Determ ination of quercetin in traditional Mongolian medicine astern by hplc. J. Of Inn. Mong. Med. Coll. 31, 571–574. 10.16343/J.Cnki.Issn.2095-512x.2009.06.007

[B93] Song N. N. Du L. J. Li X. J. Tang X. Ke J. H. (2024b). Research progress on the chemical components, pharmacological effects, and cultivation techniques of aster tataricus. South China Agric. 18, 109–115. 10.19415/J.Cnki.1673-890x.2024.15.023

[B94] Song Y. Wu Q. Jiang H. Hu A. Xu L. Tan C. (2021). The effect of shionone on sepsis-induced acute lung injury by the ecm1/stat5/nf-κb pathway. Front. Pharmacol. 12, 764247. 10.3389/Fphar.2021.764247 35153740 PMC8826228

[B95] Song Y. Wu Q. Jiang H. J. Hu A. H. Xu L. Q. Tan C. P. (2022). The effect of shionone on sepsis-induced acute lung injury by the ECM1/STAT5/NF-κB pathway. Eff. Of Shionone Sepsis-Induced Acute Lung Inj. By Ecm1/Stat5/Nf-Κb Pathw. 12, 764247. 10.3389/fphar.2021.764247 PMC8826228 35153740

[B96] Stonik V. A. Kicha A. A. Malyarenko T. V. Ivanchina N. V. (2020). Asterosaponins: structures, taxonomic distribution, biogenesis and biological activities. Mar. Drugs 18, 584. 10.3390/md18120584 33255254 PMC7760246

[B97] Su X. D. Jang H. J. Li H. X. Kim Y. H. Yang S. Y. (2019a). Identification of potential inflammatory inhibitors from aster tataricus. Bioorg Chem. 92, 103208. 10.1016/J.Bioorg.2019.103208 31473471

[B98] Su X. D. Jang H. J. Wang C. Y. Lee S. W. Rho M. C. Kim Y. H. (2019b). Anti-inflammatory potential of saponins from aster tataricus via nf-κb/mapk activation. J. Nat. Prod. 82, 1139–1148. 10.1021/Acs.Jnatprod.8b00856 30931559

[B99] Su X. D. Yang S. Y. Shrestha S. K. Soh Y. (2022). Aster saponin A(2) inhibits osteoclastogenesis through mitogen-activated protein kinase-C-Fos-Nfatc1 signaling pathway. J. Vet. Sci. 23, E47. 10.4142/Jvs.21246 35698806 PMC9346523

[B100] Sun S. M. (2019). Qian Jin Fang. Jiangsu Phoenix Science And Technology Press.

[B101] Sun Y. Li L. Liao M. Su M. Wan C. Zhang L. (2018). A systematic data acquisition and mining strategy for chemical profiling of aster tataricus rhizoma (ziwan) by uhplc-Q-tof-ms and the corresponding anti-depressive activity screening. J. Pharm. Biomed. Anal. 154, 216–226. 10.1016/J.Jpba.2018.03.022 29573734

[B102] Tanaka R. Nagao T. Okabe H. Yamauchi T. (1990). Studies On The Constituents Of Aster Tataricus L. F. Iv.: Structures Of Aster Saponins Isolated From The Herb 38, 1153–1157.

[B103] Tang X. W. Liu X. X. Tang Y. L. Liu Y. L. Xu K. H. Med J. J. T. C. V. (2006). Analysis of effective constituents from aster tataricus L. And extracting of alkaloid and its antibacterial test. Vitro 1, 16–18.

[B104] Tao H. J. (2013). Selected records of famous doctors. Beijing Traditional Chinese Medicine Press.

[B105] Tian H. Xu K. Y. Bi C. R. Hang J. S. Piao C. L. (2021). On Clinical Application And Dosage Of Tatarian Aster Root Jilin Journal Of Chinese Medicine 41, 99–102. 10.13463/J.Cnki.Jlzyy.2021.01.027

[B106] Tian R. M. Meng Y. J. Li W. Y. Ge S. J. (2012). Evaluation and analysis on germplasm resources of aster tataricus L. F. J. Of Plant Genet. Resour. 13, 984–991. 10.13430/J.Cnki.Jpgr.2012.06.012

[B107] Tian Y. P. Jing X. J. Xu L. Zhou Y. N. Zhang L. T. (2008). Determination of ferulic acid, quercetin and kaempferol in aster tataricus by hplc. Chinese traditional and herbal drugs, 926–928.

[B108] Wan C. C. Liu Y. Y. Yang H. T. Zhang Q. Y. Liao M. Zhang X. (2016). Simultaneous determination of nine constituents in asteris radix by hplc-ms/ms. Chin. Traditional And Herb. Drugs 47, 2534–2539.

[B109] Wang F. Ren G. Xiong Y. A. Zhao H. P. Yang M. (2015). Effect of shionone on il-1β,Tnf-Α and No release of macrophages induced by lipopolysaccharide. Chin. J. Of Exp. Traditional Med. Formulae 21, 123–125. 10.13422/J.Cnki.Syfjx.2015240123

[B110] Wang G. Xie W. Deng L. Huang X. Sun M. Liu W. (2024). Nrf2 mediates the effects of shionone on silica-induced pulmonary fibrosis. Chin. Med. 19, 88. 10.1186/S13020-024-00947-5 38898509 PMC11188511

[B111] Wang G. Y. Wu T. Lin P. C. Chou G. X. Wang Z. T. (2003). Phenolic compounds isolated from rhizoma of aster tataricus. China J. Of Chin. Materia Medica, 52–54. 15620184

[B112] Wang J. Liu Y. Li R. Chen H. M. Li P. (2023a). Effects of different processing methods on chemical constituents of ziwan. Pharm. And Clin. Of Chin. Materia Medica 14, 17–20+34.

[B113] Wang J. Peng S. M. Zhu S. Q. Su W. Y. Y. Q L. (2018). Ethnobotanical study on wild medicinal and edible plants in jinfo mountain region of chongqing. J. Of Plant Resour. And Environ. 27, 100–111.

[B114] Wang L. Li M. D. Cao P. P. Zhang C. F. Huang F. Xu X. H. (2014a). Astin B, a cyclic pentapeptide from Aster tataricus, induces apoptosis and autophagy in human hepatic L-02 cells. Hum. Hepatic L-02 Cells 223, 1–9. 10.1016/j.cbi.2014.09.003 25219577

[B115] Wang L. Li M. D. Lu Y. Zhang C. F. Xu X. H. Zhang M. (2014b). “Mechanism of apoptosis induced by peptide-rich fraction from the root of aster tataricus,” in Liver L-02 cells (Journal Of China Pharmaceutical University), 45, 469–474.

[B116] Wang L. Zhang M. Jin J. Huang F. Zhang C. F. (2010). Toxic fraction of radix asteris and its acute hepatotoxicity to mice. Lishizhen Med. And Materia Medica Res. 21, 2526–2528.

[B117] Wang R. Li F. R. Guo W. N. Shi X. L. (2020a). “Simultaneous determination of chlorogenic acids in,” in Aster by uplc, 33. Journal Of Wenshan University, 19–22.

[B118] Wang R. Xiao S. Niu Z. Y. (2017). Anti-cancer activity Of *Aster Tataricus* on Scc-9 human oral squamous carcinoma. Anti-Cancer Activity Of Aster Tataricus Scc-9 Hum. Oral Squamous Carcinoma 14, 142–147. 10.21010/ajtcam.v14i2.14 PMC5446437 28573230

[B119] Wang R. Zhao M. Y. Guo W. N. Cheng L. Fang C. W. (2020b). “Effects of different medicinal parts and processing methods on the content of medicinal ingredients,” in Aster, 33. Journal Of Wenshan University, 12–17.

[B120] Wang X. Fan L. Yin H. Zhou Y. Tang X. Fei X. (2020c). Protective effect of aster tataricus extract on nlrp3-mediated pyroptosis of bladder urothelial cells. J. Cell Mol. Med. 24, 13336–13345. 10.1111/Jcmm.15952 33030301 PMC7701514

[B121] Wang X. Liu Y. Li R. Chen H. M. Li P. (2023b). LncRNA BCRT1 depletion suppresses cervical cancer cell growth via sponging miR-432-5p/CCR7 axis. Tataricus. Pharmacy And Clinics Of Chinese Materia Medica 14, 17–20+34. 10.1007/s13205-023-03863-x

[B122] Wang X. Yin H. Fan L. Zhou Y. Tang X. Fei X. (2021). Shionone alleviates Nlrp3 inflammasome mediated pyroptosis in interstitial cystitis injury. Int. Immunopharmacol. 90, 107132. 10.1016/J.Intimp.2020.107132 33223465

[B123] Wang X. X. Xu H. X. Wang B. Wang X. Li Q. Meng Y. L. (2022). Effect of aster tataricus aqueous extract on pathological changes, apoptosis and expression tgf- Β1 in lung tissue of mice infected with Mycoplasma pneumoniae. Chin. J. Of Traditional Med. Sci. And Technol. 29, 975–977+1001.

[B124] Wang Y. S. (1983). Pharmacology and application of Chinese herbs, China, People’s health publishing house.

[B125] Wątroba M. Dudek I. Skoda M. Stangret A. Rzodkiewicz P. Szukiewicz D. J. A. R. R. (2017). Sirtuins, epigenetics and longevity. Epigenetics And Longev. 40, 11–19. 10.1016/j.arr.2017.08.001 28789901

[B126] Wei P. Luo Q. Hou Y. Zhao F. Li F. Meng Q. (2023). Houttuynia cordata thunb.: a comprehensive review of traditional applications, phytochemistry, pharmacology and safety. 155195. 10.1016/j.phymed.2023.155195 37956635

[B127] Wu D. Lin X. Lei S. Jiang F. Tan J. (2023). “Intervention effects of ziwan powder on airway inflammation,” in Chronic bronchitis based on tlr4/myd88/nf-κb signaling pathway, 43. Journal Of Hunan University Of Chinese Medicine, 1180–1187.

[B128] Wu H. Chen Y. Huang B. Yu Y. Zhao S. Liu J. (2021). Aster tataricus alleviates constipation by antagonizing the binding of acetylcholine to muscarinic receptor and inhibiting Ca(2+) influx. Biomed. Pharmacother. 133, 111005. 10.1016/J.Biopha.2020.111005 33378996

[B129] Wu M. Chen W. Lu Y. Zhu G. Hao L. Li Y. P. (2017). Gα13 negatively controls osteoclastogenesis through inhibition of the Akt-GSK3β-NFATc1 signalling pathway. Nat. Commun. 8, 13700. 10.1038/Ncomms13700 28102206 PMC5253683

[B130] Wu N. Huang Y. X. Shi X. Chen J. L. Peng L. F. Yang L. L. (2020). Effect of lysionotus pauciflorus maxim on signaling pathway of tlr4-myd88-nf-κb in lung tissue of rats with chronic bronchitis, 45. Journal Of Guizhou Medical University, 1283–1288. 10.19367/J.Cnki.2096-8388.2020.11.009

[B131] Wu T. Chen Z. J. Hu Y. J. Xiu Y. F. Cheng X. M. (2006). Experimental study on phlegm- resolving action of different prepared products of radix asteris. Acta Univ. Tradit. Medicalis Sin. Pharmacol., 55–57. 10.16306/J.1008-861x.2006.03.019

[B132] Wu T. Wang G. Y. Chou G. X. Gu L. H. Wang Z. T. Hu Z. B. (2003). Determination of shionone in radix asteris by hplc. Zhongguo Zhong Yao Za Zhi 28, 738–740. 15015355

[B133] Xi R. Y. Bai S. P. Sun X. D. Dong L. (2003). Determination of mineral elements and amino acids in aster tataricus L. F. Amino Acids &Biotic Resour., 8–9.

[B134] Xiu Y. F. Cheng X. M. Liu L. Wu T. Wang Z. T. (2006). Comparison of shionone content in different slices of prepared radix asteris. Acta Univ. Tradit. Medicalis Sin. Pharmacol. Shanghai, 59–61. 10.16306/J.1008-861x.2006.02.021

[B135] Xu H. M. Yi H. Zhou W. B. He W. J. Zeng G. Z. Xu W. Y. (2013). Tataricins A and B, two novel cyclotetrapeptides from Aster tataricus, and their absolute configuration assignment. And Their Absol. Configuration Assign. 54, 1380–1383. 10.1016/j.tetlet.2012.12.111

[B136] Xu M. Yang Z. Zhang W. Zhang X. (2024). Shionone improve tnbs-induced Crohn's disease like colitis by inhibiting apoptosis of intestinal epithelial cells. Journal Of Shanxi Medical University 55, 319–325. 10.13753/J.Issn.1007-6611.2024.03.007

[B137] Yang B. Xiao Y. Q. Liang R. X. Wang R. J. Li W. Zhang C. (2008). Studies on expectorant compounds in volatile oil from root and rhizome of aster tataricus. Zhongguo Zhong Yao Za Zhi 33, 281–283. 18536466

[B138] Yang D. Wang S. Huang X. Ma W. Xue Z. Zhao L. (2021). Pharmacokinetic comparison of 15 active compositions in rat plasma after oral administration of raw and honey-processed aster tataricus extracts. J. Sep. Sci. 44, 908–921. 10.1002/Jssc.202001020 33289282

[B139] Yang H. H. Son J.-K. Jung B. Zheng M. Kim J.-R. J. P. M. (2011). Epifriedelanol from the root bark of Ulmus davidiana inhibits cellular senescence in human primary cells. Hum. Prim. Cells 77, 441–449. 10.1055/s-0030-1250458 21049397

[B140] Yang T. (2003). An excellent perennial flower–aster tataricus landscape architecture academic journal, 63.

[B141] Yang X. Dai L. Yan F. Ma Y. Guo X. Jenis J. (2024). The phytochemistry and pharmacology of three rheum species: a comprehensive review with future perspectives. 155772. 10.1016/j.phymed.2024.155772 38852474

[B142] Yang Y. Y. Qin T. T. Qian S. S. Peng L. Liu L. J. Bai S. J. (2015). Effects of honey-fried radix asteris decoction(hfrad) on cell proliferation and cell cycle of colorectal cancer lovo cells. World Chin. Med. 10, 1755–1759.

[B143] Yao J. Cheng L. Zhu Y. J. Ma L. Cao Z. Z. Zhi H. (2019). Research progress on evaluation index of seed and seedling standardization system of aster tataricus. Modern agricultural science and technology, 67–68+71.

[B144] Yao P. B. Liu Y. L. Zhu Z. G. Zhao H. Zhang J. X. (2022). Effects and aster water extract on proliferation and invasion of human lung cancer A549 Cells,And tumorigenesis ability of nude mice. J. Of Pharm. Analysis 42, 380–386. 10.16155/J.0254-1793.2022.03.03

[B145] Ye J. (2007). Aster tataricus and semiaquilegia adoxoides. Master's degree, school of pharmaceutical science and technology (Tianjin University).Study on the constituents and their primary antitumor effect

[B146] Yin D. F. Zhou K. Liu J. T. Hu L. Liu Y. Deng J. (2016). Development and validation of an lc/ms/ms method for simultaneous determination of shionone and epi-friedelinol in rat plasma for pharmacokinetic study after oral administration of aster tataricus extract. Biomed. Chromatogr. 30, 1112–1117. 10.1002/Bmc.3658 26581126

[B147] Yoshioka H. Nakatsu K. Sato M. Tatsuno T. (1973). The molecular structure of cyclochlorotine,. A Toxic. Chlorine-Containing Cycl. Pentapeptide 2, 1319–1322. 10.1246/cl.1973.1319

[B148] Yu L. H. Liu G. T. (2009). The structure-activity relationship of dibenzo(A.C)cyclooctene lignans isolated from fructus schizandrae and innovation of novel ant-I hepatitis drugs progress in. Chemistry 21, 66–76.

[B149] Yu P. Cheng S. Xiang J. Yu B. Zhang M. Zhang C. (2015a). Expectorant, antitussive, anti-inflammatory activities and compositional analysis of aster tataricus. J. Ethnopharmacol. 164, 328–333. 10.1016/J.Jep.2015.02.036 25701752

[B150] Yu P. Cheng S. Xiang J. Yu B. Zhang M. Zhang C. F. (2015b). Expectorant, antitussive, anti-inflammatory activities and compositional analysis of Aster tataricus. Anti-Inflammatory Activities And Compos. Analysis Of Aster Tataricus 164, 328–333. 10.1016/j.jep.2015.02.036 25701752

[B151] Yu Z. Ma G. Liang H. (2023). Herbal textual research on the traditional Chinese medicine aster tataricuse. J. Of Chin. Med. Mater. 46, 2346–2352. 10.13863/J.Issn1001-4454.2023.09.041

[B152] Zhang G. Hao E. Xiao J. Yao C. Wang Y. Luo H. (2024). Abrus cantoniensis hance: ethnopharmacology, phytochemistry and pharmacology of A promising traditional Chinese medicine. J. Ethnopharmacol. 334, 118543. 10.1016/J.Jep.2024.118543 38986752

[B153] Zhang J. L. Jin X. Wang H. W. (2012a). Comparative analysis of volatile constituents in herbal pair flos farfarae-tatarian aster root and its single herb by gc-ms. Fine Chem. 29, 254–257. 10.13550/J.Jxhg.2012.03.005

[B154] Zhang J. W. Dou C. G. Zhang M. Ma S. P. Huang F. (2007). Toxicity of radix asteris flos farfarae and their combination. Chin. J. Of Clin. Pharmacol. And Ther., 405–411.

[B155] Zhang L. Q. Zhang Z. J. Wang X. C. Yang F. Xue C. S. (2022). Determination of shionone in roots. Stems And Flowers Of Aster By Hplc Ginseng Res. 34, 28–31. 10.19403/J.Cnki.1671-1521.2022.05.008

[B156] Zhang T. Zhou X. (2014). Clinical application of expectorant therapy in chronic inflammatory airway diseases (review). Exp. Ther. Med. 7, 763–767. 10.3892/Etm.2014.1494 24660026 PMC3961124

[B157] Zhang X. L. Meng Y. J. Jia K. X. Bai X. R. Ge S. J. (2021). Temporal and spatial variation of asterone content in aster tataricuse. Jiangsu Agric. Sci. 49, 174–179. 10.15889/J.Issn.1002-1302.2021.16.032

[B158] Zhang Y. Wang Q. Wang T. Zhang H. Tian Y. Luo H. (2012b). Inhibition of human gastric carcinoma cell growth *in vitro* by A polysaccharide from aster tataricus. Int. J. Biol. Macromol. 51, 509–513. 10.1016/J.Ijbiomac.2012.06.019 22728055

[B159] Zhao D. X. Hu B. Q. Zhang M. Zhang C. F. Xu X. H. (2015). Simultaneous separation and determination of phenolic acids, pentapeptides, and triterpenoid saponins in the root of aster tataricus by high-performance liquid chromatography coupled with electrospray ionization quadrupole time-of-flight mass Spectrometry. J. Sep. Sci. 38, 571–575. 10.1002/Jssc.201401008 25491750

[B160] Zhou W. B. (2010). Studies on chemical constituents from aster tataricus L.F. Master's degree. Hubei University Of Chinese Medicine.

[B161] Zhou W. B. Tao J. Y. Xu H. M. Chen K. L. Zeng G. Z. Ji C. J. (2010). Three New Antivir. Triterpenes Aster Tataricus 65, 1393–1396.

[B162] Zhou W. B. Zeng G. Z. Xu H. M. He W. J. Tan N. H. (2013). Astataricusones A-D and astataricusol A, five new anti-hbv shionane-type triterpenes from aster tataricus L. F. Molecules 18, 14585–14596. 10.3390/Molecules181214585 24287992 PMC6270206

[B163] Zhou W. B. Zeng G. Z. Xu H. M. He W. J. Zhang Y. M. Tan N. H. (2014). Astershionones A-F, six new anti-hbv shionane-type triterpenes from aster tataricus. Fitoterapia 93, 98–104. 10.1016/J.Fitote.2013.12.021 24393620

[B164] Zou C. Zhang R. P. Zhao B. T. Ao X. Hao X. J. Zhou J. (1999). A bioactive amide from roots of aster tartaricus acta botanica yunnanica, 123–126.

[B165] Zou W. Reeve J. L. Liu Y. Teitelbaum S. L. Ross F. P. (2008). Dap12 couples C-fms activation to the osteoclast cytoskeleton by recruitment of syk. Mol. Cell 31, 422–431. 10.1016/J.Molcel.2008.06.023 18691974 PMC2584874

