# Supplementary material for: Aster tataricus L. f.: a review on the botany, phytochemistry, ethnopharmacology, pharmacology, toxicology and comprehensive utilization
Source: Front Pharmacol. 2025 Apr 9;16:1581505. doi: 10.3389/fphar.2025.1581505 (PMC12014666; doi:10.3389/fphar.2025.1581505)
Supplement: Supplementary file 1 [file Table1.docx]

Supplementary Material

# Supplementary Tables

| **Abbreviation** | | | |
| --- | --- | --- | --- |
| BC | Before Christ | *ChP* | *Pharmacopoeia of the People's Republic of China* |
| RA | Roots of *A.tataricus* |  |  |
| ICP-MS | Inductively coupled plasma-Mass Spectrometry | U | Uranium |
| Ca | Calcium | Mg | Magnesium |
| Zn | Zinc | Mn | Manganese |
| Fe | Ferrum | Cu | Cuprum |
| Mo | Molybdenum | V | Vanadium |
| Cr | chromium | Ga | Gallium |
| Ag | Argentum | Cd | cadmium |
| Tl | Titanium | SHS | A Single Shionone Synthase |
| NRPS | Nonribosomal peptide synthetase | NO | Nitric Oxide |
| PGE-2 | Prostaglandin E2 | TNF-α | tumor Necrosis Factor Alpha |
| IL-1β | **Interleukin-1beta** | IL-6 | Interleukin- 6 |
| LPS | lipopolysaccharide | NF-ĸB | Nuclear Factor kappa-B |
| iNOS | Inducible Nitric Oxide Synthase | COX-2 | Cyclooxygenase-2 |
| JNK | c-Jun N-terminal kinase | P38 | P38 mitogen-activated protein kinase |
| 4-HPA | 4-Hydroxyphenylacetic acid | HIF-1α | Hypoxia-inducible factor |
| NLRP3 | Nucleotide- binding oligomerization domain, Leucine- Rich repeat and Pyrin domain- containing 3 | ASC | A Caspase Recruitment Domain |
| Caspase | Cysteinyl aspartate specific proteinase | ROS | Reactive Oxygen Species |
| TLR4 | Toll-Like Receptor 4 | STZ | Streptozotocin |
| OVA | Ovalbumin | RANKL | Receptor Activator of Nuclear Factor Kappa-B Ligand |
| ACh | A**cetylcholine** | M-CSF | Macrophage Colony Stimulating Factor |
| ATE | *A.tataricus* extract | BMM | Bone Marrow Macrophages |
| VEGF | Vascular Endothelial Growth Factor | ERK | Extracellular Regulated Protein Kinases |
| CAT | catalase | AChR | A**cetylcholine Receptor** |
| GSH | Glutathione peroxidase | SOD | Superoxide dismutase |
| Nrf2 | Nuclear factor erythroid 2-related factor 2 | ATEE | the ethanol extract of *A.tataricus* |
